# Health economics evidence for food system policy: a systematic review of reviews

**DOI:** 10.3389/fnut.2025.1629814

**Published:** 2025-12-12

**Authors:** Lin Fu, Irina Pokhilenko, Thijs van Rens, Richard Smith, Emma Frew

**Affiliations:** 1 Centre for Economics of Obesity, School of Applied Health Sciences, College of Medicine and Health, University of Birmingham, Birmingham, United Kingdom; 2 Department of Economics, University of Warwick, Coventry, United Kingdom; 3 Department of Public Health and Sport Sciences, Faculty of Health and Life Sciences, University of Exeter, Exeter, United Kingdom

**Keywords:** economics, health, food-system, cost-effectiveness, costs

## Abstract

Food system interventions are needed for improving public health and to address health inequalities. Economics evidence enables an understanding of the costs associated with these interventions when offset against the gains to society. Here, we define economic analysis as studies of the effect of an intervention on prices, profits or balance sheets. By mapping this evidence, we gain an understanding of whether interventions target the production (supply-side) or consumption (demand-side) aspects of the system. This is helpful for identifying which “cluster” of policies have been more readily investigated in the academic literature, what the evidence says, and where there are gaps in evidence. The aims of this review were to synthesise the economics evidence on food system interventions designed to improve population health, to identify the most studied areas of the system, the economics methods used, and any trends in methodological choices based on the type of intervention. A search strategy to identify reviews published between 2012 and 2024 was applied to seven databases. The focus was on the whole population in high-income countries. Thirty-six reviews were identified covering 718 primary studies. There were 21 reviews focused on demand-side interventions, such as taxes and subsidies on sugar-sweetened beverages or foods high in salt, sugar and fat, and only four reviews that looked at solely supply-side interventions. Eleven reviews were on a mix of both demand and supply side interventions. We found that supply side (e.g., reformulation) interventions were more effective at altering dietary behaviours when compared to demand side interventions (e.g., labelling). Supply-side evaluations tended to use ex-ante methods for hypothetical interventions, while demand side evaluations used a mix of both ex-ante and ex-post designs. Synthesising the cost-effectiveness of the interventions across the reviews was challenging due to heterogeneity of methods applied. Due to the wide range of agents and sectors involved, a cost–benefit analysis from a societal perspective is the most recommended economic evaluation method. Further studies are needed to identify the most efficient mix of intervention components likely to achieve substantial health and economic impacts.

**Systematic review registration:**

https://www.crd.york.ac.uk/prospero/, Identifier CRD42022360714.

## Introduction

Over the last 20 years there has been increasing evidence on how the food system is contributing to worsening population health. Factors such as the increased supply of ultra-processed foods (1), the emergence of the convenience culture (2), the influence of marketing campaigns promoting foods high in fat, sugar and salt (HFSS) (3), social norms (4), and decline in home cooking skills (5), have been reported as among the causes. This has led to governments globally paying greater attention to intervening in the food system and developing food system strategies such as the UK National Food Strategy in 2021 (6), the Canadian Food Policy in 2019 (7), the EU Farm to Fork Strategy in 2020 (8), and the Australian National Food Plan in 2013 (9).

Economics is key when assessing which food system policies to implement. First, there is a need to understand the economic principles and processes within the system without any intervention, establishing the relationships and processes determining how food is produced, distributed, and consumed. Second, there is a need to understand the economics associated with potential interventions, including their cost-effectiveness (which interventions will bring about the greatest benefit for least cost), how the costs and benefits are distributed across different ‘agents’ in the system, the timing of costs and benefits (including how short term investment might lead to longer term population health gains) and how investing in different parts of the food system can lead to gains or losses in other parts of the system.

Challenging methodological issues emerge when evaluating food system interventions as they are often complex in nature, occur in real-world settings, involve multiple stakeholders and sectors, and have several interacting components. This requires adapting methods to take account of this complexity and the use of both qualitative and quantitative techniques to answer a wider range of research questions (10). Economics plays an important role within complex evaluation design, as it can help answer questions related to the scalability and sustainability of interventions and ensures an understanding of the distribution of costs and benefits across the whole food system.

Despite recognising the significance of the economics of food system interventions, there is a notable gap in understanding how that has shaped research activities and influenced the distribution of economics evidence across various types of interventions. Identifying which interventions have received the most attention in academic studies helps to reveal key research gaps and what the evidence indicates regarding the effectiveness of interventions from an economics perspective. Furthermore, it can offer insights into the trends in methods used within the research studies, as well as any patterns related to where in the food system these interventions are implemented. It can also highlight whether specific types of interventions are associated with particular methodological evaluative approaches.

This paper reports a timely review of existing reviews to facilitate a more systematic understanding of the economics of food system interventions, in terms of the quantity, type, and quality of the economics evidence available. The focus is on high-income country settings as these countries have food systems with a set of features including technological advancements, consumer behaviours, and health and nutrition challenges, very different to low- and middle-income country settings. It is also focused on identifying evidence for interventions that have a clear pathway to understanding impact upon population health.

## Materials and methods

### Protocol and registration

The review followed the Preferred Reporting Items for Systematic Reviews and Meta-Analyses (PRISMA) guidelines, and a narrative synthesis was conducted. A protocol was developed and registered in PROSPERO (International Prospective Register of Systematic Reviews, registration number CRD42022360714).

### Search strategy

Table 1 reports on the modified PICOS (population, intervention, comparison, outcome and study design) framework that was applied. Only systematic literature review papers were included. Reviews were eligible for inclusion if they reported on the economic analysis of a food system intervention designed to improve population health. The definition of “economic analyses” of food system interventions was limited to financial impacts only, i.e., the intervention design resulted in a change to the product price (e.g., taxes and subsidies) or the analysis considered impacts of the intervention on household budgets, food industry profit or balance sheets; the search also considered reviews of economic evaluations of food system interventions. The search was restricted to reviews published in the English language between 1st January 2012 and 4th November 2024, on the basis that any review published since 2012 will include primary studies from pre-2012. Additionally, forward and backward citation searching was applied. The review was focused on evaluations of food system intervention that considered population health impact, so considerations of solely environmental impact were excluded.

**Table 1:** PICOS criteria.

Criteria	Included	Excluded
Population	Reviews that included general population within high-income country setting	Population with only low or middle-income country settings. Children only population.
Intervention	Food system interventions	Interventions not relating to food
Comparison	Any relevant comparator within the food system	Not applicable
Outcome	Explicit or implicit references to health outcomes	Reviews of studies that do not report health outcomes
Study type	Peer-reviewed systematic reviews published between 2012 and 2024. Reviews published in English language	Original primary studies, conference abstracts, study protocols, grey literature, scoping reviews.
Economic analysis	Reviews of economic evaluations; reviews of interventions that had a fiscal property (tax, subsidy, change to price); or reviews that considered the fiscal impact of interventions, e.g., change to price, profit, household budgets.	Qualitative studies. Any reviews that were of primary studies with no consideration of the fiscal impact (either within the intervention, or upon costs or outcomes).

Seven health and economic databases were searched: MEDLINE (Ovid), EMBASE (Ovid), CINAHL Plus, EconLit, PsycINFO, Cochrane Library, Centre for Reviews and Dissemination (CRD). The search strategy identified papers that included at least one search term in each of four sets of keywords around food/diet, interventions/policy, population health, and economic analysis, see Table 2 for details. To validate the search strategy, 39 relevant papers known to the authors were identified in advance and subsequently confirmed they had been identified. A list of these 39 papers is available in Supplementary File, Table S1 and a list of search terms for all databases is available in Table S2.

**Table 2:** Keywords organised into four categories.

Components	Keywords
Food	Sugar; sweetened; junk; beverage; carbonated beverage; salt; sodium; trans fatty acids; dietary fats; fatty acids; fruit; fruit and vegetable juice; vegetable; food; diet; food supply; food growing; agriculture; supermarket; restaurant; food preference
Interventions	Intervention; tax; price; government programs; policy; government regulation; formulated food; subsidy; advertising; marketing; promotion; labelling; nutrition information; nutrition warning; health promotion; levy; nutrition policy; nutritional requirements; financing; legislation; change; decrease; reduce; limit; modify; new; reformulation; restriction; replace; increase; discount; deal; offer; ban
Population	Population; national population; government; population health; global health; public health; policymaker; consumer; food industry
Economics analysis	Economic analysis; macroeconomic; economic evaluation; cost; cost–benefit analysis; cost consequence analysis; difference in difference; interrupted time series analysis; regression analysis; model; computer simulation; natural experiment; demand analysis; economic; econometric; QALY; DALY; cross-sectional studies

### Eligibility criteria

All duplicates were removed using Endnote (version 21) software. The records then underwent a two-stage screening approach (11). First, the titles and abstracts were independently screened by two authors (LF and IP) based on the eligibility as described by PICOS. Second, the full texts were retrieved and reviewed by LF and IP using the PICOS criteria, and any reasons for exclusion were documented. Any disagreements between the reviewers were resolved by discussion.

### Data extraction and analysis

To facilitate a narrative synthesis of all included reviews, data was first extracted into a table with the following themes: the focus of intervention and whether it targeted the demand or supply-side of the food system; the study design (such as cost–benefit analysis; interrupted time-series); the measure of effectiveness; the types of costs considered; whether distributional effects had been considered; and the timing of the evaluation, e.g., if the policy had already been implemented in real-life or was a hypothetical policy being modelled.

For classification of demand- or supply-side interventions, we applied the following criteria, consistent with conventional economic theory. Demand-side interventions included fiscal policies such as taxes imposed primarily at the point of consumption, as well as subsidies aimed at altering household income. Supply-side interventions were defined as those targeting food producers or manufacturers, including policies that incentivise production or reformulation of specific food products. A notable exception to this classification was the sugar-sweetened beverage (SSB) tax. While the SSB tax is commonly implemented as an excise tax at the point of purchase in countries like Hungary, Denmark, France, Finland, Mexico, Portugal and Ireland, thus aligning with the demand-side approach, the UK has introduced a sugar levy targeting producers to encourage reformulation, which fits with a supply-side approach. Since it was not feasible to examine every primary study within each SSB-tax review, we classified SSB tax reviews broadly as demand-side interventions, reflecting the prevalence of point-of-purchase SSB tax globally. Where possible, evidence specific to the UK’s supply side sugar levy was highlighted separately.

With respect to the study design, the reviews of economic evaluations were reported separately as a specific type of economic analysis that is commonly used within health economics to estimate how “non-health” (food system) interventions impact on population health. These reviews were analysed to determine how costs and outcomes were incorporated, and in particular if, and how, non-health costs were included, as food-system interventions are likely to have costs that fall outside the health care sector.

In addition, general review characteristics were documented, such as the country setting, the population included, the stated aim of the review, the number of primary studies included, and quality assessment results (if available). Data extraction was performed by the main reviewer (LF) with a proportion checked (20%) by IP.

### Quality/risk of bias assessment

To assess the quality of the systematic reviews two methods were applied. First, and due to the expected heterogeneity in study design and methods applied, appraisal of the evidence relied on the review authors assessment using the relevant quality appraisal tool detailed in the Results section. Second, the A MeaSurement Tool to Assess systematic Reviews (AMSTAR 2) was applied (12). This is an adapted version of the AMSTAR to give more prominence to risk of bias within non-randomised studies. Only the critical domains (determined by the AMSTAR 2 developers) were assessed independently by two reviewers (LF and IP). Any disagreement between the reviewers were resolved by discussion with all authors. Note this review included all reviews that passed the inclusion/exclusion criteria, and the quality assessment using AMSTAR 2 were not considered as a criterion for inclusion.

To help with the synthesis of all the reviews identified, the Graphical Representation of Overlap for OVErviews (GROOVE) was also used (13). This tool produces an overall corrected covered area which is an assessment of the degree of overlap of primary studies included within the reviews. For more detail on this tool, please refer to Bracchiglione et al. (13).

## Results

### Study selection

**Figure 1:**
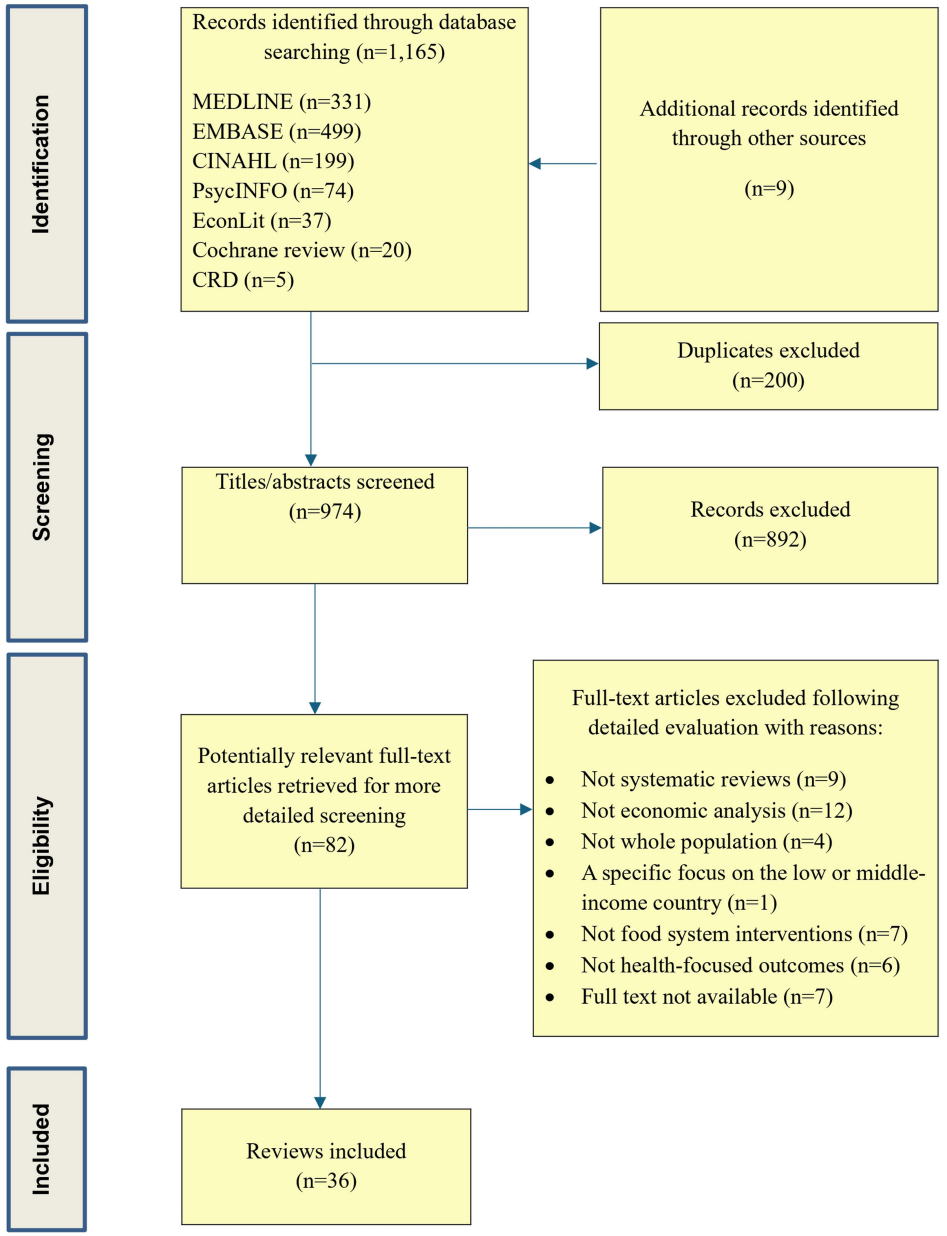
PRISMA flowchart.

The database search identified 1,165 reviews, and nine additional reviews were identified from forward and backward citation checking. After removal of duplicates, the title and abstracts were screened. A full text screening was conducted on 82 reviews, and 36 reviews were included. The PRISMA diagram (Figure 1) documents each stage of the selection process.

### Quality/risk of bias assessment

Review authors used different criteria to judge the quality of the included studies, detailed in Table S3. These were often applied according to the primary study design, e.g., Cochrane risk of bias tool for interrupted time series analysis. For the economic evaluations, several methods were used to assess quality, including the Consensus on Health Economic Criteria CHEC-list (14), the CHEERs checklist (15–20), the Drummond checklist (21), and the British Medical Journal checklist (22). Across all of the reviews, the quality varied extensively. Either the review authors did not evaluate the quality of the primary studies (eight reviews), or the quality was reported to be high (17–19, 23), moderate (24), or low (14, 15, 21, 22, 25–29). Fourteen reviews reported narratively that the quality was variable by study design, methods, and outcomes (16, 20, 30–41). There were no clear trends of quality of evidence when looking at the demand versus the supply-side of the food system.

Using the AMSTAR 2 appraisal tool revealed consistent limitations with how risk of bias was assessed within the included reviews (Table 3). A common limitation was failure to include a complete list of potentially relevant studies with justification for exclusion of each one (item 7), and with taking account of risk of bias in individual studies when summarising the results (item 9). Item 15 did not apply to many reviews because most lacked quantitative synthesis, and this item concerns the investigation of publication bias and its effect on syntheses.

**Table 3:** Quality assessment of reviews using the AMSTAR 2 critical domains (12).

Quality assessment	AMSTAR 2 critical domains (items)						
Authors, year	Item 2	Item 4	Item 7	Item 9	Item 11	Item 13	Item 15
Thow et al. (2014) (25)	No	Partial yes	No	No	N/A	No	N/A
Maniadakis et al. (2013) (42)	No	Partial yes	No	No	N/A	Yes	N/A
Hyseni et al. (2017) (39)	Partial yes	Partial yes	No	No	N/A	No	N/A
Redondo et al. (43) (2018)	No	Partial yes	No	Partial yes	N/A	Yes	N/A
Wyse et al. (2021) (44)	Partial yes	Partial yes	No	Yes	Yes	Yes	No
von Philipsborn et al. (2019) (40)	Yes	Yes	Yes	Yes	Yes	Yes	Yes
Tran et al. (2021) (20)	Partial yes	Partial yes	No	No	N/A	No	N/A
Teng et al. (2019) (30)	Partial yes	Partial yes	Yes	Yes	Yes	Yes	Yes
Schorling et al. (2017) (22)	No	Partial yes	No	No	N/A	No	N/A
Powell et al. (2013) (45)	No	Partial yes	No	No	N/A	No	N/A
Pfinder et al. (2020) (26)	Yes	Yes	Yes	Yes	N/A	Yes	N/A
Olm et al., 2020 (18)	No	Partial yes	No	Yes	N/A	Yes	N/A
Niebylski et al. (2015) (24)	No	Partial yes	No	No	N/A	No	N/A
McLaren et al. (2016) (28)	Yes	Yes	Yes	Yes	yes	Yes	Yes
Liu et al. (2022) (19)	No	Partial yes	No	No	N/A	No	N/A
Lhachimi et al. (2020) (27)	Yes	Yes	Yes	Yes	N/A	No	N/A
Hope et al. (2017) (17)	No	Partial yes	Yes	No	N/A	Yes	N/A
Hillier-Brown et al. (2017) (41)	Partial yes	Partial yes	Yes	No	N/A	No	N/A
Gittelsohn et al. (2017) (31)	No	Partial yes	No	No	N/A	No	N/A
Fattore et al. (2014) (14)	No	Partial yes	No	No	N/A	No	N/A
Eyles et al. (2012) (32)	No	Partial yes	No	No	N/A	Yes	N/A
Epstein et al. (2012) (46)	No	Partial yes	No	No	N/A	No	N/A
Emmert-Fees et al. (2021) (16)	Partial yes	Partial yes		No	N/A	Yes	N/A
Dangour et al. (2013) (23)	No	Partial yes	No	No	N/A	No	N/A
De Steur et al. (2017) (47)	No	Partial yes	No	No	yes	Yes	No
Cornelsen et al. (2015) (37)	No	Partial yes	No	No	yes	No	No
Cormick et al. (2021) (15)	Yes	Partial yes	No	Yes	yes	Yes	No
Barberio et al. (2017) (29)	No	Yes	No	Yes	N/A	No	Yes
Afshin et al. (2017) (33)	No	Partial yes	No	No	yes	No	Yes
Aguiar et al. (2017) (21)	No	Partial yes	No	No	N/A	No	N/A
Alagiyawanna et al. (2015) (38)	No	Yes	No	Yes	N/A	N/A	Yes
Alcaraz et al. (2021) (48)	No	Yes	No	No	N/A	N/A	N/A
An (2013) (34)	No	Partial yes	Yes	Yes	N/A	Yes	N/A
Backholer et al. (2016) (35)	No	Yes	No	No	N/A	No	N/A
Pineda et al. (2024) (36)	Yes	Partial yes	No	Yes	N/A	Yes	N/A
Thiboonboon et al. (2024) (49)	No	Partial yes	Yes	No	N/A	No	N/A

Item 2: Did the review contain an explicit statement that the review methods were established prior to conduct of the review and justify any significant deviations from the protocol?; Item 4: Did the authors use a comprehensive literature search strategy?; Item 7: Did the authors provide a list of excluded studies and justify the exclusions?; Item 9: Did the authors use a satisfactory technique for assessing the risk of bias (RoB) in individual studies that were included in the review?; Item 11: If meta-analysis was justified did the authors use appropriate methods for statistical combination of results?; Item 13: Did the authors account for RoB in individual studies when interpreting/ discussing the results?; Item 15: If they performed quantitative synthesis did they carry out an adequate investigation of publication bias and discuss likely impact on the results?; N/A: not applicable.

For the degree of overlap, we assessed the 36 reviews covering 718 primary studies using the GROOVE tool. The ‘overall corrected cover area’ was 1.35%, which is considered a slight overlap between the 36 reviews and unlikely to influence any conclusions. With only a slight overlap within the “overall corrected cover area”, this meant we could proceed to assess the quantity of evidence based on the total number of primary studies contained within each review. The primary study counts therefore are “non-additive” as a single primary study may have appeared in multiple systematic reviews.

### Study characteristics

The reviews were published between 2012 and 2024 with the majority published since 2017. The volume of included primary studies within each review ranged widely, from one to 78 primary studies. The reviews referred to food systems within different country settings with the USA being the most frequently studied country (included within 33 reviews), followed by Australia (24 reviews) and the UK (23 reviews). All the reviews were focused on whole population interventions without any restriction by population subgroup. All the review authors reported no conflict of interest, including no competing interests or reporting any funding received for conducting the review. The main characteristics of the included reviews are presented in Table S3 and summarised in Table 3.

**Figure 2:**
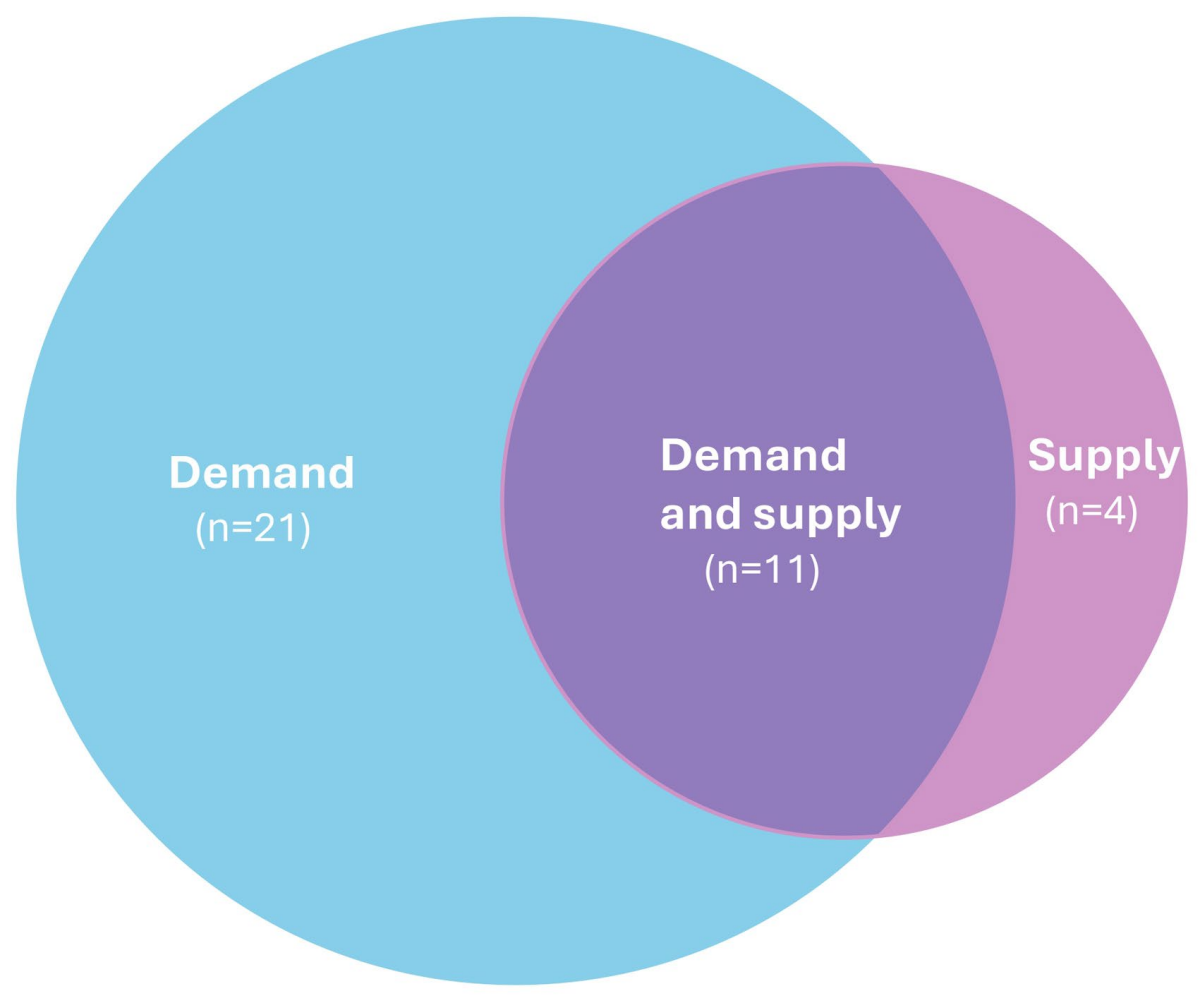
Distribution of evidence across food system.

Figure 2 illustrates how the evidence within the reviews was categorised between the demand or supply side of the food system. Below in the Table 4, we give a more detailed breakdown of the types of interventions that were included within each category, with full details provided in Table S3.

### Supply side reviews

The four reviews identified as being on the supply-side included 54 primary studies. Three reviews focused on reformulation and one review on agricultural pricing policy. Evidence within these reviews were synthesised using narrative synthesis, and meta-analysis for a subset of primary studies. All four reviews translated the effects of interventions into health outcomes, covering disease-specific outcomes (such as falls prevention and weight-related metrics) as well as quality-adjusted life years (QALYs) and disability-adjusted life years (DALYs). Only one review on reformulation reported that genetically modified biofortified crops, targeting vitamin A, folate, iron, or zinc, could improve micronutrient intake and reduce the aggregated national-level micronutrient deficiency burden in a cost-effective way (47). However, the other reviews on agricultural pricing policy, calcium fortification, and vitamin D deficiency prevention strategies reported the cost-effectiveness evidence to be unclear (15, 21, 23).

### Demand side reviews

A large number of reviews were identified that focused on interventions targeting the demand-side of the food system, with a total of 21 reviews covering 584 primary studies (14, 19, 24–27, 30–38, 42–46, 49). The majority of these reviews (19 reviews) focused on fiscal policy, including taxes (nine reviews), subsidies (one review) and both taxes and subsidies (nine reviews), measuring their effects on changes to purchasing or consumption. Fourteen of these reviews translated these behavioural changes to health outcomes, such as daily nutrient intake, body weight, QALYs, DALYs, and life years gained.

The specific types of tax policy included SSB tax (four reviews), fat tax (one review), SSB and fat tax (one review), sugar tax (two reviews), or HFSS tax (one review).

The nine reviews that considered both tax and subsidy policy in combination covered 364 primary studies. Although many of these reviews reported good evidence on own-price elasticity, a common finding was lack of evidence on cross-price elasticity effects, so a lack of understanding on how the tax/subsidy on the targeted good would impact purchasing of non-targeted goods. This led to an overall conclusion that the evidence for the effectiveness of these policies (on health) was inconclusive. For example, the largest review in this category that covered 78 primary studies reported that a tax/subsidy that altered prices by a minimum of 10–15% would be effective at improving dietary behaviours that could impact overall health, however, the failure to capture substitution effects with non-taxed products could potentially undermine this effectiveness (24). This point was echoed by the review on SSB and HFSS tax, which reported a lack of evidence on how these taxes would impact overall calorie intake and subsequent weight outcomes (42). Other notable findings from these reviews on fiscal policy were the importance of controlling for dynamic social environments and the wider country context, as the generalisability of fiscal policy will depend on the influence of these external factors.

Four reviews of SSB tax covered 60 primary studies, and concluded that SSB tax is effective at reducing purchases of SSBs, with a meta-analyses from the largest review reporting a SSB tax elasticity of −1.00 (95% CI -0.50 to −1.47) (30) Only 1 of the 60 primary studies was on the UK sugar levy and the review authors did not differentiate results between the UK sugar levy and SSB tax implemented in other country settings.

### Mix of demand and supply-side reviews

Eleven reviews covering 369 primary studies were of interventions across both the demand- and supply-sides of the food system. The largest category (five reviews) focused on salt reduction, pulling together evidence for multi-component strategies that included reformulation, labelling, pricing and procurement. The remaining reviews covered a mix of interventions to reduce SSB consumption (40, 48), various nutritional interventions for reducing population obesity (18), a review of health-promoting retail-based interventions (20), and on how to promote healthier ready meals (41), and a review of model-based economic evaluations on reformulation, tax and labelling (16).

In terms of effectiveness, most (eight reviews) measured the effectiveness of interventions by analysing purchase data, and this was particularly the case for salt and SSB-reduction strategies. Seven of these reviews then translated the effects into health outcomes, covering disease-specific outcomes (such as hypertension and cardiovascular disease prevention) as well as QALYs, DALYs, and life years gained.

The largest category of reviews (on salt reduction policy) covered 156 primary studies, with the largest review within this category synthesising evidence from 70 primary studies (39). The review concluded that a comprehensive strategy, involving upstream interventions would achieve the largest salt reduction in the population. The importance of including upstream interventions was also noted by two further reviews in this category (28, 29), and one review mentioned that reformulation needed to be mandatory rather than voluntary to achieve the largest gains (22). All reviews agreed that a multi-component strategy comprising multiple interventions offered the most effective approach for reducing salt in diets because of synergy effects (17, 28, 29, 39).

The next largest category of reviews was on SSB reduction policies. We identified two reviews covering 98 primary studies. Although with low to moderate confidence in the certainty of effects, one of these reviews found that interventions that altered the environment in which people make beverage choices had the strongest effect. These interventions included restricting availability of SSBs in schools, labelling, altering the default option in children’s menus, product positioning in the retail environment, and restricting purchasing of SSBs within government-led food benefit programmes (40). The other review was of model-based economic evaluations covering 40 primary modelling studies. They found the cost-effectiveness evidence for SSB reduction policy to be inconclusive, mainly due to the wide range of modelling methods used and the lack of evidence for translating change in SSB purchasing to health outcomes (48).

Four other reviews were identified that covered both the demand- and the supply-side of the food system. These covered nutritional interventions to reduce risk of obesity; retail-based interventions; economic evidence for healthier ready meals; and a review of economic evaluations for a combination of reformulation, tax and food labelling. All of these reviews reported on low-agency interventions being the most effective, most reported on the clear benefits of making changes to the food system for health, however there was notable uncertainty around accounting for potential compensating behaviours and lack of attention to the whole diet within the evaluation of interventions that targeted specific foods (20).

### Study design

**Figure 3:**
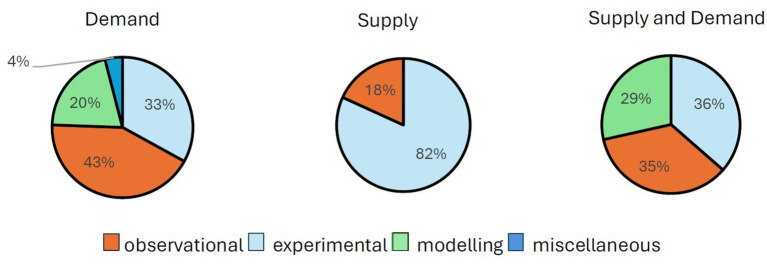
Primary study design distributed across the food system.

To understand how the primary study design varied across the food system, we categorised the primary studies (excluding reviews of economic evaluations which are reported in full below) contained within the reviews. Figure 3 illustrates the distribution, categorised into: experimental designs including randomised controlled trials, quasi-experimental, non-randomised trials, interrupted time series analysis; or observation studies including cross-sectional studies; or decision-analytic modelling studies; or miscellaneous designs as described by the review authors. Reviews of economic evaluations were grouped into a separate category and included cost–benefit, cost-utility and cost-effectiveness analysis.

We were also interested in understanding how the distribution of evidence fared with respect to policies that had already been implemented, versus hypothetical policies (for which the effects could be modelled). Interestingly, almost all the reviews of evidence for supply-side interventions were ex-ante evaluations, specifically looking at vitamin D and calcium fortification/supplementation, and agricultural policy. For the demand-side, these contained a mix of evaluations with some being ex-ante (six reviews), others ex-post (three reviews), and eight reviews containing a mixture of both. As expected for the combined reviews that covered both demand and supply side interventions (11 reviews), these contained a mix of ex-ante (five reviews) and ex-post interventions (two reviews), and a combination of both (four reviews). There were no clear trends in study design for hypothetical versus already implemented interventions.

### Distributional effects

Ensuring access to healthy affordable food for all in society is an overarching aim for any national food strategy. We were therefore keen to identify the reviews of food system interventions that attempted to synthesise the evidence with respect to impact on dietary inequalities, and to understand more around the methods and data the review authors used to estimate these distributional effects.

The importance of capturing the distributional impact was reported by most review authors, across all the interventions. However, a common challenge was lack of evidence from the primary studies to understand the effects with any certainty. Where possible, the review authors reported effects by subgroup, for example the impact of calcium fortification on children and adults separately (15), or salt reduction interventions for men compared to women (28, 29). Some of the reviews of pricing policy reported a greater impact on lower socioeconomic groups (19, 35, 36, 43, 45). Many review authors reported on the need for interventions to be upstream and low agency to reduce inequalities in diet, but most of these statements were incorporated into the discussion rather than quantitatively estimated. Although it was recognised that food system interventions have an important role to play with tackling dietary inequalities, the evidence for measuring the distributional impact was not available to enable review authors to synthesise with certainty.

### Reviews of economic evaluations

We identified 10 reviews that focused solely on economic evaluation evidence comprising 208 primary studies. Two reviews were of supply-side interventions (21, 47), three were for demand-side interventions (14, 19, 49), and the remaining five reviews were for multi-component interventions covering both supply and demand interventions (16–18, 20, 22). The majority of the reviews (seven reviews) of economic evaluations were based on ex-ante modelling studies, which lowered the certainty of the review conclusions.

There was no clear pattern of type of economic evaluation used according to type of intervention. Across all economic evaluations, there was a range of effectiveness data synthesised including purchasing data, nutritional intake and dietary outcomes for the cost-effectiveness analysis, and health outcomes modelled into QALYs or DALYs for cost-utility analyses. It was interesting to note that of the 208 primary studies included across the 10 reviews, only two reviews included a cost–benefit analysis (CBA) (14, 47), where one review explored the return on investment from genetically modifying food and the other estimated the cost-effectiveness of voluntary interventions (labelling and counselling) aimed at promoting low-fat diets. The largest review of economic evaluations (*n* = 56 primary studies) was restricted to model-based economic evaluations for reformulation, tax and labelling, and noted the lack of models that included economic effects beyond the health sector (16). With the exception of one review (47), all other nine reviews (including 192 economic evaluations) reported on the perspective for capturing intervention costs. Within these nine reviews, either a health care (*n* = 59 primary studies) or a societal perspective (*n* = 58), was the most common. We found only five reviews (14, 16, 17, 22, 49) that mentioned the cost to the food industry, e.g., product reformulation and administration burden.

Many of the reviews reported unclear evidence for cost effectiveness (14, 17, 20–22, 49), with a common challenge being the lack of empirical data to populate model parameters, leaving modellers to often rely on assumptions (14, 49). This was noted in the review of economic evaluations for salt reduction interventions with a recommendation that results from primary modelling studies should only be compared after the models are carefully checked for comparability as the heterogeneity in modelling methods makes comparisons of cost-effectiveness challenging (22).

## Discussion

To the best of our knowledge, this is the first review of reviews exploring economic evidence for food system interventions. We document that: (1) relative to the supply-side of the food system, more evidence is available for interventions that target the demand-side (mostly focussed on fiscal policy designed to alter demand for food/drinks): (2) economic analysis of food system interventions adopt a wide range of methods applied within different study designs; and (3) review authors often report on inconclusive results due to the inability to capture ‘whole of diet’ impacts, or lack of long-term data to extrapolate to health outcomes.

We found only 10 reviews reporting evidence from economic evaluation studies. There was a high level of methodological heterogeneity making synthesis of results challenging. Of note was the lack of evidence from CBAs, and limited evidence therefore on the wider costs and outcomes across the various agents and sectors involved in a food system. We found various types of costs included across the different studies. Less than half of primary studies within the reviews of economic evaluations considered the societal perspective, and only half of the reviews included costs to the food industry.

The lack of evidence on food-system interventions, particularly on supply-side interventions, is exacerbated by the fact that many studies evaluate hypothetical policies rather than policies that have already been implemented, a lack of evidence to capture distributional impact, and widely varying quality of primary studies. Particularly within the economic evaluation evidence, a large proportion of modelling assumptions were required to extrapolate long-term effects which increased the uncertainty of evidence. Of particular note was the lack of data on cross-price elasticity and therefore “whole of diet” effects, making extrapolation to long term outcomes highly uncertain. The importance of accounting for mediating factors such as food insecurity and income inequality when considering the impact of food pricing policy was noted in a recent empirical study, finding that the underlying income distribution across the population had a direct causal impact on how a change in food prices affects long term health system expenditure (50). None of the reviews of economic evaluations considered the potential for these mediating factors. Also,the heterogeneity in terms of the type of intervention, type of food, study design (modelling, experiments, empirical studies), setting (e.g., supermarket, restaurants, vending machine) and economic analysis methods used also challenged the synthesis of (cost-) effectiveness results. As a result various quality appraisal tools were used, which makes the comparison of quality of studies not possible. Use of the AMSTAR2 quality appraisal tool revealed that it was rare for reviews to explicitly account for risk of bias in individual studies included in their review, lacking consideration of the potential for confounding, and the potential for bias of exposures and outcomes, e.g., using purchasing data as a surrogate for consumption.

### Strengths and limitations

A strength of this review is that it used a comprehensive search strategy, validated through identifying ‘known relevant papers’ in advance to check they had been found by the strategy. All title/abstracts and full texts of papers were screened independently by two authors. We synthesised this wide and diverse body of evidence by categorising evaluations into either the demand-side or supply-side of the food system, enabling a more comprehensive understanding of where the evidence lies, how methods differ, and cost-effectiveness.

The key limitation of the study is that the eligibility criteria were limited to the reviews that implicitly or explicitly considered the impact of interventions on health and those interventions that alter the price of foods, household budgets, corporate profits or balance sheets. Therefore, our review most likely does not capture the full breadth of economic evidence in relation to food system interventions. Our demand-side classification included SSB-tax as being imposed at the point of consumption noting the limitation that there are examples of other tax designs that are more supply-focused, such as the UK sugar levy. Where possible we highlighted the primary studies focused on the UK sugar levy separately but we may have missed some primary studies. In addition, our review was focused on whole population interventions in high-income countries and English language reviews, which may have led to some evidence being excluded. Nevertheless, we believe that our synthesis of economic considerations in relation to health will offer valuable evidence to support the design of viable and sustainable food policies.

Furthermore, we synthesised the evidence assuming only minimal overlap of primary studies. Although we applied the GROOVE tool and found only a slight overlap between the 36 reviews, there will likely have been some duplications of primary studies across the reviews. In terms of the economic modelling studies, it was not always clear if statistical modelling, econometric modelling, or decision-analytic modelling had been applied, making it difficult to draw conclusions.

In terms of future research, we recommend that more focus is given to interventions that alter the supply-side of the food system, an area which is currently lacking in evidence. Examples could include offering business tax-relief for companies who have a healthier offer, bringing together small-medium enterprise (SME) business owners to create networks and offer training, providing grant funding for technical innovation. We recognise that this will require working with the food industry and political leadership as noted in the recent UK House of Lords Select Committee Report on Food, Diet, and Obesity (51). We also suggest that a similar review of reviews like this one is undertaken for LMIC. We also recommend that when evaluating food-system interventions using economic evaluation, that authors carefully consider the full range of relevant costs, including those that fall onto industry, and that where possible, the analysis explicitly takes account of potential dietary substitution effects and resulting impact on health outcomes. All analysis need to pay attention to heterogeneous effects and distributional impacts where possible, and ex-post evaluations completed for food-system interventions that are implemented. There is also a need for review authors to attempt to assess quality of the primary studies and use this assessment to inform their findings. Finally, we note that several reviews highlighted stronger effects from multiple interventions than single interventions because of synergistic effects (17, 28, 29, 39). Future studies are needed to identify the most effective mix of intervention components, and in what populations and settings.

## Conclusion

This review of reviews provides a comprehensive understanding of the economic evidence for food system interventions, highlighting key trends, gaps, and methodological considerations. While the demand-side of the food system has received more attention, particularly regarding fiscal policies to influence consumer behaviour, there was a notable lack of empirical evidence on long term health outcomes, unintended dietary impacts, and distributional consequences. Economic evaluations varied widely in methods, scope and quality, making synthesis challenging and often reliant on assumptions.

Our findings suggest that more robust and comprehensive economic evaluations are needed, especially incorporating societal and industry perspectives, and those targeting the under-research supply side of the food system. Future research should also explore synergistic effects of multiple interventions, identify the most effective combinations across diverse populations and settings, and ensure methodological clarity and transparency in modelling approaches.

**Table 4:** Summary of included reviews.

Author/year (number of primary studies in review)	Intervention focus
Supply side interventions (4 reviews)	
Dangour et al., 2013 (23) (*n* = 4)	Agricultural policy (direct effect on food price)
De Steur et al., 2017 (47) (*n* = 16)	Agricultural reformulation (genetic modification)
Cormick et al., 2021 (15) (*n* = 20)	Calcium fortification
Aguiar et al., 2017 (21) (*n* = 14)	Vitamin D deficiency prevention
Demand side interventions (21 reviews)	
Thow et al., 2014 (25) (*n* = 38)	Food tax and subsidies
Redondo et al., 2018 (43) (*n* = 17)	Sugar-sweetened beverage (SSB) tax
Maniadakis et al., 2013 (42) (*n* = 55)	Tax on SSBs or Foods High in Fat Sugar or Salt (HFSS)
Backholer et al., 2016 (35) (*n* = 11)	Tax on SSBs
Teng et al., 2019 (30) (*n* = 18)	“Real world” SSB tax
Thiboonboon et al., 2024 (49) (*n* = 14)	Tax on SSBs
Powell et al., 2013 (45) (*n* = 36)	Food and beverage tax and subsidies
Pfinder et al., 2020 (26) (*n* = 1)	Tax of unprocessed sugar or sugar-added foods
Niebylski et al., 2015 (24) (*n* = 78)	Food subsidies and tax
Liu et al., 2022 (19) (*n* = 15)	Tax on sugary foods and beverages
Lhachimi et al., 2020 (27) (*n* = 2)	Fat tax on foods
Gittelsohn et al., 2017 (31) (*n* = 30)	Pricing policy using tax or subsidies
Eyles et al., 2012 (*n* = 32) (32)	Pricing policy using tax or subsidies
Cornelsen et al., 2015 (37) (*n* = 78)	Pricing policy using tax, price or subsidies
Afshin et al., 2017 (33) (*n* = 30)	Pricing policies using tax, price or subsidies
Alagiyawanna et al., 2015 (38) (*n* = 18)	Tax and subsidies
Pineda et al., 2024 (36) (*n* = 20)	Tax on HFSS
Epstein et al., 2012 (46) (*n* = 24)	Pricing policy
An, 2013 (34) (*n* = 20)	Healthy food subsidies
Wyse et al., 2021 (44) (*n* = 11)	Mix of online interventions
Fattore et al., 2014 (14) (*n* = 36)	Voluntary interventions promoting low-fat diet
Demand and supply side interventions (11 reviews)	
Hyseni et al., 2017 (39) (*n* = 70)	Salt reduction
Schorling et al., 2017 (22) (*n* = 14)	Salt reduction
McLaren et al., 2016 (28) (*n* = 17)	Salt reduction
Hope et al., 2017 (17) (*n* = 14)	Salt reduction
Barberio et al., 2017 (29) (*n* = 41)	Salt reduction
von Philipsborn et al., 2019 (40) (*n* = 58)	SSB reduction
Alcaraz et al., 2021 (48) (*n* = 40)	SSB reduction
Tran et al., 2021 (20)(*n* = 8)	Mix of health-promoting retail-based interventions
Olm et al., 2020 (18) (*n* = 21)	Obesity policies: including nutritional interventions
Hillier-Brown et al., 2017 (41) (*n* = 30)	Mix of interventions to promote healthier ready meals
Emmert-Fees et al., 2021 (16) (*n* = 56)	Mix of population-based dietary policies
