# Supplementary material for: Health economics evidence for food system policy: a systematic review of reviews
Source: Front Nutr. 2025 Dec 12;12:1629814. doi: 10.3389/fnut.2025.1629814 (PMC12742207; doi:10.3389/fnut.2025.1629814)
Supplement: Supplementary file 1 [file Table_1.DOCX]

Supplementary Material

# Supplementary Table S1

1. Lock K, Smith RD, Dangour A, Keogh-Brown M, Pigatto G, Hawkes C, Fisjberg R, Chalabi Z. Health, Agricultural and Economic Effects of Adopting Healthy Diet Recommendations. *The Lancet, 2010; 376: 1699-1709*.
2. Green R, Cornelsen L, Turner R, Dangour AD, Shankar B, Mazzocchi M, RD Smith. The effect of rising food prices on food consumption: systematic review with meta-regression.  *British Medical Journal, 2013;* *346:f3703 doi: 10.1136/bmj.f3703.*
3. Cornelsen L, Green R, Turner R, Dangour AD, Shankar B, Mazzocchi M, RD Smith. What happens to patterns of food consumption when food prices change? Evidence from a systematic review and meta-analysis of food price elasticities globally. *Health Economics, 2015; 24: 1548-1559*.
4. Shemilt I, Marteau TM, Smith RD, Ogilvie D.  Use and cumulation of evidence from modelling studies to inform policy on food taxes and subsidies: biting off more than we can chew? *BMC Public Health, 2015; 15: 297. DOI 10.1186/s12889-015-1641-5.*
5. Cornelsen L, Green R, Dangour AD, Mazzocchi M, RD Smith. Estimating the relationship between food prices and food consumption – methods matter. *Applied Economic Perspectives* *and Policy, 2016; 38 (3): 546-561*.
6. Cornelsen L, Mytton O, Adams J, Gasparrini A, Iskander D, Knai C, Petticrew M, Scott C, Smith R, Thompson C, White M, Cummins S.  Change in non-alcoholic beverage sales following a 10-pence levy on sugar-sweetened beverages within a national chain of restaurants in the UK: interrupted time series analysis of a natural experiment.  *Journal of Epidemiology and Community Health, 2017; 71: 1107–1112. Doi: 10.1136/jech-2017-209947*
7. Tuomisto HL, Scheelbeek PFD, Chalabi Z, Green R, Smith RD, Haines A, Dangour AD. Effects of environmental change on population nutrition and health: a comprehensive framework with a focus on fruits and vegetables. *Wellcome Open Research, 2017; 2:21. doi: 10.12688/wellcomeopenres.11190.2*.
8. Quirmbach D, Cornelsen L, Jebb S, Marteau T, Smith RD. Effect of increasing the price of sugar-sweetened beverages on alcoholic beverage purchases: an economic analysis of sales data.  *Journal of Epidemiology and Community Health, 2018; 72: 324–330.* [*http://dx.doi.org/10.1136/jech-2017-209791*](http://dx.doi.org/10.1136/jech-2017-209791)*.*
9. Smith RD, Cornelsen L, Quirmbach D, Jebb S, Marteau T. Reducing sugar consumption: are sweet snacks more sensitive to price increases than sugar-sweetened beverages?  *BMJ Open 2018;8:e019788. doi:10.1136/bmjopen-2017-019788.*
10. Walls HL, Johnston D, Tak M, Dixon J, Hanefeld J, Hull E, Smith RD.  The impact of agricultural input subsidies on food and nutrition security: A systematic review.  *Food Security, 2018; 10(6):1425–1436.* [*https://doi.org/10.1007/s12571-018-0857-5*](https://doi.org/10.1007/s12571-018-0857-5)*.*
11. Cuevas S, Cornselsen L, Smith RD, Walls H. Economic globalization, nutrition and health: a review of quantitative evidence.  *Globalization and Health, 2019; 15:15.* [*https://doi.org/10.1186/s12992-019-0456-z*](https://doi.org/10.1186/s12992-019-0456-z).
12. Cornelsen L, Mazzocchi M, Smith RD. Fat tax or thin subsidy? How price increases and decreases affect the energy and nutrient content of food and beverage purchases in Great Britain.  *Social Science & Medicine, 2019.* [*https://doi.org/10.1016/j.socscimed.2019.04.003*](https://doi.org/10.1016/j.socscimed.2019.04.003).
13. Scheelbeek P, Cornelsen L, Marteau TM, Jebb SA, Smith RD.  Potential impact on prevalence of obesity in the UK of a 20% price increase in high sugar snacks: modelling study.  *British Medical Journal, 2019; 366: l4786.* [*http://dx.doi.org/10.1136/bmj.l4786*](http://dx.doi.org/10.1136/bmj.l4786)*.*
14. Jensen HT, Keogh-Brown M, Shankar B, Basu S, Cuevas S, Dangour A, Gheewala SH, Green R, Joy E, Thaiprasert N, Smith RD. Palm oil and dietary change: Application of an integrated macroeconomic, environmental, demographic, and health modelling framework for Thailand. *Food Policy, 2019; 83: 92-103.* [*https://doi.org/10.1016/j.foodpol.2018.12.003*](https://doi.org/10.1016/j.foodpol.2018.12.003)*.*
15. Jensen HT, Keogh-Brown M, Shankar B, Aekplakorn W, Basu S, Cuevas S, Dangour A, Gheewala SH, Green R, Joy E, Rojroongwasinkul N, Thaiprasert N, Smith RD. International trade, dietary change, and cardiovascular disease health outcomes: Import tariff reform using an integrated macroeconomic, environmental and health modelling framework for Thailand. *Social Science & Medicine – Population Health, 2019: 100435.* [*https://doi.org/10.1016/j.ssmph.2019.100435*](https://doi.org/10.1016/j.ssmph.2019.100435).
16. Keogh-Brown MR, Jensen HT, Basu S, Aekplakorn W, Cuevas S, Dangour A, Gheewala SH, Green R, Joy E, Rojroongwasinkul N, Thaiprasert N, Shankar B, Smith RD. Evidence on the magnitude of the economic, health and population effects of palm cooking oil consumption: An integrated modelling approach with Thailand as a case study.*Population Health Metrics, 2019; 17:12.* [*https://doi.org/10.1186/s12963-019-0191-y*](https://doi.org/10.1186/s12963-019-0191-y)*.*
17. Cornelsen L, Quaife M, Lagarde M, Smith RD. Framing and signaling effects of taxes on sugary beverages: A Discrete Choice Experiment among households in Great Britain.  *Health Economics, 2020; 29: 1132-1147. DOI: 10.1002/hec.4123*.
18. Law C, Cornelsen L, Adams J, Penney T, Rutter H, White M, Smith RD. An analysis of the stock market reaction to the announcements of the UK Soft Drinks Industry Levy.  *Economics and Human Biology, 38: August 2020.* [*https://doi.org/10.1016/j.ehb.2019.100834*](https://doi.org/10.1016/j.ehb.2019.100834)*.*
19. Law C, Cornelsen L, Adams J, Pell D, Rutter H, White M, Smith RD.  The impact of UK Soft Drinks Industry Levy on manufacturers’ domestic turnover.  *Economics and Human Biology, 2020.* [*http://dx.doi.org/10.1016/j.ehb.2020.100866*](http://dx.doi.org/10.1016/j.ehb.2020.100866)*.*
20. Biondi B, Cornelsen L, Mazzocchi M, Smith RD. Between preferences and references: asymmetric price elasticities and the simulation of fiscal policies. *Journal of Economic Behaviour and Organization, 2020; 180: 108-128.* [*https://doi.org/10.1016/j.jebo.2020.09.016*](https://doi.org/10.1016/j.jebo.2020.09.016)*.*
21. Watt T, Beckert W, Smith RD, Cornelsen L.  Reducing consumption of unhealthy foods and beverages through banning price promotions: what is the evidence and will it work?  *Public Health Nutrition, 2020.* [*https://doi.org/10.1017/S1368980019004956*](https://doi.org/10.1017/S1368980019004956)*.*
22. Pell D, Penney TL, Mytton O, Briggs A, Cummins S, Rayner M, Rutter H, Scarborough P, Smith RD, White M, Adams J. Anticipatory changes in British household purchases of soft drinks associated with the announcement of the Soft Drinks Industry Levy: a controlled interrupted time series analysis.  *PLOS Medicine, 2020; 17(11) e1003269:*[*https://doi.org/10.1371/journal.pmed.1003269*](https://doi.org/10.1371/journal.pmed.1003269).
23. Carriedo A, Koon A, Lee K, Ooms G, Silva D, Smith J, Smith RD, Walls H (alphabetical authorship).  Political economy of sugar sweetened beverages: Perspectives on taxation in Latin America.  *Globalization and Health, 2021; 15 (5):*[*https://doi.org/10.1186/s12992-020-00656-2*](https://doi.org/10.1186/s12992-020-00656-2)
24. Matita1 M, Chirwa EW, Johnston D, Mazalale J, Smith RD, Walls H. Does household participation in food markets increase dietary diversity? Evidence from rural Malawi. *Global Food Security, 2021; 28: 100486*[*https://doi.org/10.1016/j.gfs.2020.100486*](https://doi.org/10.1016/j.gfs.2020.100486)*.*
25. Pell D, Mytton O, Penney TL, Briggs A, Cummins S, Pedder-Jones C, Rayner M, Rutter H, Scarborough P, Smith RD, White M, Adams J. Changes in soft drinks purchased by British households associated with the UK soft drinks industry levy: controlled interrupted time series analysis.  *British Medical Journal, 2021; 372:*[*http://dx.doi.org/10.1136/bmj.n254*](http://dx.doi.org/10.1136/bmj.n254).
26. Milsom P, Smith RD, Modisenyane M, Walls H.  Do international trade and investment agreements generate regulatory chill in public health policymaking? A case study of nutrition and alcohol policy in South Africa. *Globalization and Health, 2021;*[*https://doi.org/10.1186/s12992-021-00757-6*](https://doi.org/10.1186/s12992-021-00757-6).
27. Milsom P, Smith RD, Baker P, Walls H.  International investment liberalization, transnational corporations and NCD prevention policy non-decisions: A realist review on the political economy of tobacco, alcohol and ultra-processed food.  *Globalization and Health, 2021; 17: 134.* [*https://doi.org/10.1186/s12992-021-00784-3*](https://doi.org/10.1186/s12992-021-00784-3).
28. Law C, Smith RD, Cornelsen L. Place matters: out-of-home demand for foods and drinks in Great Britain.  *Food Policy, 2022:*[*https://doi.org/10.1016/j.foodpol.2021.102215*](https://doi.org/10.1016/j.foodpol.2021.102215)*.*
29. Milsom P, Walls H, Baker P, Smith RD.  Corporate power and the international trade regime as drivers of NCD policy non-decisions: A realist review.  *Health Policy and Planning (in press)* [*https://doi.org/10.1093/heapol/czaa148*](https://doi.org/10.1093/heapol/czaa148).
30. Yau, A., Berger, N., Law, C., Cornelsen, L., Greener, R., Adams, J., Boyland, E.J., Burgoine, T., de Vocht, F., Egan, M. and Er, V., 2022. Changes in household food and drink purchases following restrictions on the advertisement of high fat, salt, and sugar products across the Transport for London network: A controlled interrupted time series analysis. PLoS medicine, 19(2), p.e1003915.
31. Liu, S., Veugelers, P.J., Liu, C. and Ohinmaa, A., 2021. The Cost Effectiveness of Taxation of Sugary Foods and Beverages: A Systematic Review of Economic Evaluations. Applied health economics and health policy, pp.1-14.
32. Sacks, G., Veerman, J.L., Moodie, M. and Swinburn, B., 2011. ‘Traffic-light’nutrition labelling and ‘junk-food’tax: a modelled comparison of cost-effectiveness for obesity prevention. International journal of obesity, 35(7), pp.1001-1009.
33. Du, M., Griecci, C.F., Cudhea, F.F., Eom, H., Kim, D.D., Wilde, P., Wong, J.B., Wang, Y.C., Michaud, D.S., Mozaffarian, D. and Zhang, F., 2021. Cost-effectiveness Analysis of Nutrition Facts Added-Sugar Labeling and Obesity-Associated Cancer Rates in the US. JAMA network open, 4(4), pp.e217501-e217501.
34. Cobiac, L.J., Tam, K., Veerman, L. and Blakely, T., 2017. Taxes and subsidies for improving diet and population health in Australia: a cost-effectiveness modelling study. PLoS medicine, 14(2), p.e1002232.
35. Lal, A., Mantilla-Herrera, A.M., Veerman, L., Backholer, K., Sacks, G., Moodie, M., Siahpush, M., Carter, R. and Peeters, A., 2017. Modelled health benefits of a sugar-sweetened beverage tax across different socioeconomic groups in Australia: A cost-effectiveness and equity analysis. PLoS medicine, 14(6), p.e1002326.
36. Collins, B., Capewell, S., O’Flaherty, M., Timpson, H., Razzaq, A., Cheater, S., Ireland, R. and Bromley, H., 2015. Modelling the health impact of an english sugary drinks duty at national and local levels. PLoS One, 10(6), p.e0130770.
37. Allen, K., Pearson-Stuttard, J., Hooton, W., Diggle, P., Capewell, S. and O’Flaherty, M., 2015. Potential of trans fats policies to reduce socioeconomic inequalities in mortality from coronary heart disease in England: cost effectiveness modelling study. Bmj, 351.
38. Collins, M., Mason, H., O’Flaherty, M., Guzman-Castillo, M., Critchley, J. and Capewell, S., 2014. An economic evaluation of salt reduction policies to reduce coronary heart disease in England: a policy modeling study. Value in Health, 17(5), pp.517-524.
39. Briggs, A.D., Mytton, O.T., Kehlbacher, A., Tiffin, R., Rayner, M. and Scarborough, P., 2013. Overall and income specific effect on prevalence of overweight and obesity of 20% sugar sweetened drink tax in UK: econometric and comparative risk assessment modelling study. Bmj, 347.

**Supplementary Table S2: Search strategy for different databases**

| **Medline – 331 hits**  **1.** Sugars/  2. (sweeten* or junk).tw.  3. Dietary Sugars/  4. Beverages/  5. Carbonated Beverages/  6. 1 or 2 or 3 or 4 or 5  7. Salts/  8. sodium, dietary/  9. 7 or 8  10. *Trans Fatty Acids/  11. *Dietary Fats/  12. *Fatty Acids/  13. Fruit/  14. "Fruit and Vegetable Juices"/  15. Vegetables/  16. Food/  17. food*.tw.  18. Diet/  19. Food Supply/  20. Food growing.tw.  21. Agriculture/  22. *supermarkets/  23. *Restaurants/  24. Food Preferences/  25. 6 or 9 or 10 or 11 or 12 or 13 or 14 or 15 or 16 or 17 or 18 or 19 or 20 or 21 or 22 or 23 or 24  26. *Taxes/  27. (tax* or price* or pricing).tw.  28. Government Programs/ or Policy/ or Government Regulation/  29. Food, Formulated/  30. (change or changing or decreas* or limit* or modify or modified or new or reduce or reducing or reduction* or reformulat* or restrict* or replac* or increas* or discount or deal* or offer* or ban).tw.  31. subsid*.tw.  32. Advertising/  33. (market* or adverti* or promot*).tw.  34. Food Labelling/  35. ((food* or nutrition* or diet*) adj10 (facts or information or label* or symbol* or warning*)).tw.  36. Health Promotion/  37. levy.tw.  38. Nutrition Policy/  39. *Nutritional Requirements/  40. Financing, Government/  41. Legislation, Food/  42. interven*.tw.  43. 26 or 27 or 28 or 29 or 30 or 31 or 32 or 33 or 34 or 35 or 36 or 37 or 38 or 39 or 40 or 41 or 42  44. population.tw.  45. exp Adult/  46. Government/  47. national population.tw.  48. Population Health/  49. Global Health/  50. Public Health/  51. (policy maker* or policymaker*).tw.  52. Consumer*.tw.  53. customer*.tw.  54. purchas*.tw.  55. consumption.tw.  56. Food Industry/  57. 44 or 45 or 46 or 47 or 48 or 49 or 50 or 51 or 52 or 53 or 54 or 55 or 56  58. "economic analysis".mp.  59. macroeconomic*.mp.  60. macro-economic*.mp.  61. "economic evaluat*".mp.  62. cost*.ti.  63. "cost benefit analys*".mp.  64. Cost-Benefit Analysis/  65. "costs and cost analysis"/  66. "cost consequence".mp.  67. "difference in difference".mp.  68. Interrupted Time Series Analysis/  69. Regression Analysis/  70. Models, Theoretical/  71. Computer Simulation/  72. models, statistical/  73. "natural experiment".mp.  74. "demand analysis".mp.  75. "discrete choice model*".mp.  76. "discrete choice experiment".mp.  77. *Economics/  78. Econometric*.mp.  79. Models, Economic/  80. quality-adjusted life years/  81. Disability-Adjusted Life Years/  82. Cross-Sectional Studies/  83. 58 or 59 or 60 or 61 or 62 or 63 or 64 or 65 or 66 or 67 or 68 or 69 or 70 or 71 or 72 or 73 or 74 or 75 or 76 or 77 or 78 or 79 or 80 or 81 or 82  84. 25 and 43 and 57 and 83  85. review*.ti.  86. 84 and 85  87. limit 86 to english language  88. limit 87 to humans  89. limit 88 to yr="2012 -Current" |
| --- |
| **Embase – 499**  **1.** sugar/  2. (sweeten* or junk).tw.  3. sugar intake/  4. beverage/  5. carbonated beverage/  6. 1 or 2 or 3 or 4 or 5  7. sodium chloride/  8. sodium intake/  9. 7 or 8  10. *trans fatty acid/  11. *fat intake/  12. *fatty acid/  13. fruit/  14. "Fruit and Vegetable Juices".tw.  15. vegetable/  16. food/  17. food*.tw.  18. diet/  19. catering service/  20. food growing.tw.  21. agriculture/  22. *supermarket/  23. *restaurant/  24. food preference/  25. 6 or 9 or 10 or 11 or 12 or 13 or 14 or 15 or 16 or 17 or 18 or 19 or 20 or 21 or 22 or 23 or 24  26. *tax/  27. (tax* or price* or pricing).tw.  28. policy/ or government regulation/  29. elemental diet/  30. (change or changing or decreas* or limit* or modify or modified or new or reduce or reducing or reduction* or reformulat* or restrict* or replac* or increas* or discount or deal* or offer* or ban).tw.  31. subsid*.tw.  32. advertising/  33. (market* or adverti* or promot*).tw.  34. food packaging/  35. ((food* or nutrition* or diet*) adj10 (facts or information or label* or symbol* or warning*)).tw.  36. health promotion/  37. levy.tw.  38. nutrition policy/  39. *nutritional requirement/  40. public finance/  41. food legislation/  42. interven*.tw.  43. 26 or 27 or 28 or 29 or 30 or 31 or 32 or 33 or 34 or 35 or 36 or 37 or 38 or 39 or 40 or 41 or 42  44. population/  45. population.tw.  46. adult/  47. government/  48. national population.tw.  49. population health/  50. global health/  51. public health/  52. (policy maker* or policymaker*).tw.  53. Consumer*.tw.  54. customer*.tw.  55. purchas*.tw.  56. consumption.tw.  57. food industry/  58. 44 or 45 or 46 or 47 or 48 or 49 or 50 or 51 or 52 or 53 or 54 or 55 or 56 or 57  59. "economic analysis".mp.  60. macroeconomic*.mp.  61. macro-economic*.mp.  62. "economic evaluat*".mp.  63. cost*.ti.  64. "cost benefit analys*".mp.  65. “cost benefit analysis”/  66. "costs and cost analysis"/  67. "cost consequence".mp.  68. "difference in difference".mp.  69. "Interrupted Time Series".tw.  70. regression analysis/  71. theoretical model/  72. computer simulation/  73. statistical model/  74. "natural experiment".mp.  75. "demand analysis".mp.  76. "discrete choice model*".mp.  77. "discrete choice experiment".mp.  78. *economics/  79. Econometric*.mp.  80. economic model/  81. quality adjusted life year/  82. disability-adjusted life year/  83. cross-sectional study/  84. 59 or 60 or 61 or 62 or 63 or 64 or 65 or 66 or 67 or 68 or 69 or 70 or 71 or 72 or 73 or 74 or 75 or 76 or 77 or 78 or 79 or 80 or 81 or 82 or 83  85. 25 and 43 and 58 and 84  86. review*.ti.  87. 85 and 86  88. limit 87 to english language  89. limit 88 to human  90. limit 89 to yr="2012 -Current" |
| **PsycInfo =>74**   1. Sugars/ 2. (sweeten* or junk).tw. 3. “Dietary Sugar”.tw. 4. “Beverages (Nonalcoholic)”/ 5. “Carbonated Beverage”.tw. 6. 1 or 2 or 3 or 4 or 5 7. Salt.tw. 8. Food Intake/ 9. 7 or 8 10. fatty acids/ 11. Fruit/ 12. "Fruit and Vegetable Juices"/ 13. Vegetables/ 14. Food/ 15. food*.tw. 16. diets/ 17. Food Supply/ 18. Food growing.tw. 19. Agriculture/ 20. Retailing/ 21. Food Preferences/ 22. 6 or 9 or 10 or 11 or 12 or 13 or 14 or 15 or 16 or 17 or 18 or 19 or 20 or 21 23. Taxation/ 24. (tax* or price* or pricing).tw. 25. Government Programs/ or Policy Making/ 26. (change or changing or decreas* or limit* or modify or modified or new or reduce or reducing or reduction* or reformulat* or restrict* or replac* or increas* or discount or deal* or offer* or ban).tw. 27. subsid*.tw. 28. Advertising/ 29. (market* or adverti* or promot*).tw. 30. “Food Labelling”.tw. 31. ((food* or nutrition* or diet*) adj10 (facts or information or label* or symbol* or warning*)).tw. 32. Health Promotion/ 33. levy.tw. 34. Nutrition Policy/ 35. Nutritional Requirements/ 36. Financing, Government/ 37. Legislation, Food/ 38. interven*.tw. 39. 23 or 24 or 25 or 26 or 27 or 28 or 29 or 30 or 31 or 32 or 33 or 34 or 35 or 36 or 37 or 38 40. Population/ 41. adult*.tw. 42. population.tw. 43. Government/ 44. national population.tw. 45. Population Health/ 46. Global Health/ 47. Public Health/ 48. (policy maker* or policymaker*).tw. 49. Consumer*.tw. 50. customer*.tw. 51. purchas*.tw. 52. consumption.tw. 53. Food Industry/ 54. 40 or 41 or 42 or 43 or 44 or 45 or 46 or 47 or 48 or 49 or 50 or 51 or 52 or 53 55. "economic analysis".mp. 56. macroeconomic*.mp. 57. macro-economic*.mp. 58. "economic evaluat*".mp. 59. cost*.ti. 60. "cost benefit analys*".mp. 61. Cost-Benefit Analysis/ 62. "costs and cost analysis"/ 63. "cost consequence".mp. 64. "difference in difference".mp. 65. “Interrupted Time Series”.tw. 66. Statistical Regression/ 67. models/ 68. Computer Simulation/ 69. models, statistical/ 70. "natural experiment".mp. 71. "demand analysis".mp. 72. "discrete choice model*".mp. 73. "discrete choice experiment".mp. 74. Economics/ 75. Econometric*.mp. 76. “quality-adjusted life years”.tw. 77. “Disability-Adjusted Life Years”.tw. 78. “Cross-Sectional”.tw. 79. 55 or 56 or 57 or 58 or 59 or 60 or 61 or 62 or 63 or 64 or 65 or 66 or 67 or 68 or 69 or 70 or 71 or 72 or 73 or 74 or 75 or 76 or 77 or 78 80. 22 and 39 and 54 and 79 81. review*.ti. 82. 80 and 81 83. limit 82 to english language 84. limit 83 to human 85. limit 84 to yr="2012 -Current" |
| **EconLit – 37**   1. TI Sugar* OR AB Sugar* 2. TI ( sweeten* or junk ) OR AB ( sweeten* or junk ) 3. TI (Beverage* OR Drink*) OR AB (Beverage* OR Drink*) 4. TI (Carbonated Beverage* OR Soft Drink*) OR AB (Carbonated Beverage* OR Soft Drink*) 5. S1 OR S2 OR S3 OR S4 6. TI Salt* OR AB Salt* 7. TI Trans Fatty Acids OR AB Trans Fatty Acids 8. TI Dietary Fat* OR AB Dietary Fat* 9. TI Fatty Acids OR AB Fatty Acids 10. TI Fruit* OR AB Fruit* 11. TI ( Fruit and Vegetable Juice* ) OR AB ( Fruit and Vegetable Juice* ) 12. TI Vegetable* OR AB Vegetable* 13. TI food* OR AB food* 14. TI Diet* OR AB Diet* 15. TI food growing OR AB food growing 16. TI supermarket* OR AB supermarket* 17. TI Restaurant* OR AB Restaurant* 18. S5 OR S6 OR S7 OR S8 OR S9 OR S10 OR S11 OR S12 OR S13 OR S14 OR S15 OR S16 OR S17 19. TI tax* OR AB tax* 20. TI ( price* or pricing ) OR AB ( price* or pricing ) 21. TI Formulated Food OR AB Formulated Food 22. TI ( change or changing or decreas* or limit* or modify or modified or new or reduce or reducing or reduction* or reformulat* or restrict* or replac* or increas* or discount or deal* or offer* or ban ) OR AB ( change or changing or decreas* or limit* or modify or modified or new or reduce or reducing or reduction* or reformulat* or restrict* or replac* or increas* or discount or deal* or offer* or ban ) 23. TI subsid* OR AB subsid* 24. TI Advertising OR AB Advertising 25. TI ( market* or adverti* or promot* ) OR AB ( market* or adverti* or promot* ) 26. TI Food Labeling OR AB Food Labeling 27. TI ( (food* or nutrition* or diet*) AND (fact* or information or label* or symbol* or warning*) ) OR AB ( (food* or nutrition* or diet*) AND (fact* or information or label* or symbol* or warning*) ) 28. TI Health Promotion OR AB Health Promotion 29. TI levy OR AB levy 30. TI Food Legislation* OR AB Food Legislation* 31. TI Interven* OR AB Interven* 32. S19 OR S20 OR S21 OR S22 OR S23 OR S24 OR S25 OR S26 OR S27 OR S28 OR S29 OR S30 OR S31 33. TI Population OR AB Population 34. TI Adult* OR AB Adult* 35. TI Government OR AB Government 36. TI national population OR AB national population 37. TI ( policy maker* or policymaker* ) OR AB ( policy maker* or policymaker* ) 38. TI Consumer* OR AB Consumer* 39. TI customer* OR AB customer* 40. TI purchas* OR AB purchas* 41. TI consumption OR AB consumption 42. TI manufacturer* OR AB manufacturer* 43. S33 OR S34 OR S35 OR S36 OR S37 OR S38 OR S39 OR S40 OR S41 OR S42 44. TI "economic analysis" OR AB "economic analysis" 45. TI macroeconomic* OR AB macroeconomic* 46. TI macro-economic* OR AB macro-economic* 47. TI "economic evaluat*" OR AB "economic evaluat*" 48. TI "cost benefit analys*" OR AB "cost benefit analys*" 49. TI Cost-Benefit Analysis OR AB Cost-Benefit Analysis 50. TI costs and cost analysis OR AB costs and cost analysis 51. TI "cost consequence" OR AB "cost consequence" 52. TI "difference in difference" OR AB "difference in difference" 53. TI "Interrupted Time Series" OR AB "Interrupted Time Series" 54. TI Regression* OR AB Regression* 55. TI "natural experiment" OR AB "natural experiment" 56. TI "demand analysis" OR AB "demand analysis" 57. TI "discrete choice experiment" OR "discrete choice model*" OR AB "discrete choice experiment" OR "discrete choice model*" 58. TI Economic* OR AB Economic* 59. TI Econometric* OR AB Econometric* 60. TI "quality-adjusted life years" OR AB "quality-adjusted life years" 61. TI "Disability-Adjusted Life Years" OR AB "Disability-Adjusted Life Years" 62. TI cross-sectional analysis OR AB cross-sectional analysis 63. S44 OR S45 OR S46 OR S47 OR S48 OR S49 OR S50 OR S51 OR S52 OR S53 OR S54 OR S55 OR S56 OR S57 OR S58 OR S59 OR S60 OR S61 OR S62 64. S18 AND S32 AND S43 AND S63 65. TI review* 66. S64 AND S65 67. Limiters - Published Date: 20120101-; Publication Type: Journal Article 68. Narrow by Language: - english |
| **CINAHL Plus - 199**  **1.** TI Sugar* OR AB Sugar*  2. TI ( sweeten* or junk ) OR AB ( sweeten* or junk )  3. TI (Beverage* OR Drink*) OR AB (Beverage* OR Drink*)  4. TI (Carbonated Beverage* OR Soft Drink*) OR AB (Carbonated Beverage* OR Soft Drink*)  5. S1 OR S2 OR S3 OR S4  6. TI Salt* OR AB Salt*  7. TI Trans Fatty Acids OR AB Trans Fatty Acids  8. TI Dietary Fat* OR AB Dietary Fat*  9. TI Fatty Acids OR AB Fatty Acids  10. TI Fruit* OR AB Fruit*  11. TI ( Fruit and Vegetable Juice* ) OR AB ( Fruit and Vegetable Juice* )  12. TI Vegetable* OR AB Vegetable*  13. TI food* OR AB food*  14. TI Diet* OR AB Diet*  15. TI food growing OR AB food growing  16. TI supermarket* OR AB supermarket*  17. TI Restaurant* OR AB Restaurant*  18. S5 OR S6 OR S7 OR S8 OR S9 OR S10 OR S11 OR S12 OR S13 OR S14 OR S15 OR S16 OR S17  19. TI tax* OR AB tax*  20. TI ( price* or pricing ) OR AB ( price* or pricing )  21. TI Formulated Food OR AB Formulated Food  22. TI ( change or changing or decreas* or limit* or modify or modified or new or reduce or reducing or reduction* or reformulat* or restrict* or replac* or increas* or discount or deal* or offer* or ban ) OR AB ( change or changing or decreas* or limit* or modify or modified or new or reduce or reducing or reduction* or reformulat* or restrict* or replac* or increas* or discount or deal* or offer* or ban )  23. TI subsid* OR AB subsid*  24. TI Advertising OR AB Advertising  25. TI ( market* or adverti* or promot* ) OR AB ( market* or adverti* or promot* )  26. TI Food Labeling OR AB Food Labeling  27. TI ( (food* or nutrition* or diet*) AND (fact* or information or label* or symbol* or warning*) ) OR AB ( (food* or nutrition* or diet*) AND (fact* or information or label* or symbol* or warning*) )  28. TI Health Promotion OR AB Health Promotion  29. TI levy OR AB levy  30. TI Food Legislation* OR AB Food Legislation*  31. TI Interven* OR AB Interven*  32. S19 OR S20 OR S21 OR S22 OR S23 OR S24 OR S25 OR S26 OR S27 OR S28 OR S29 OR S30 OR S31  33. TI Population OR AB Population  34. TI Adult* OR AB Adult*  35. TI Government OR AB Government  36. TI national population OR AB national population  37. TI ( policy maker* or policymaker* ) OR AB ( policy maker* or policymaker* )  38. TI Consumer* OR AB Consumer*  39. TI customer* OR AB customer*  40. TI purchas* OR AB purchas*  41. TI consumption OR AB consumption  42. TI manufacturer* OR AB manufacturer*  43. S33 OR S34 OR S35 OR S36 OR S37 OR S38 OR S39 OR S40 OR S41 OR S42  44. TI "economic analysis" OR AB "economic analysis"  45. TI macroeconomic* OR AB macroeconomic*  46. TI macro-economic* OR AB macro-economic*  47. TI "economic evaluat*" OR AB "economic evaluat*"  48. TI "cost benefit analys*" OR AB "cost benefit analys*"  49. TI Cost-Benefit Analysis OR AB Cost-Benefit Analysis  50. TI costs and cost analysis OR AB costs and cost analysis  51. TI "cost consequence" OR AB "cost consequence"  52. TI "difference in difference" OR AB "difference in difference"  53. TI "Interrupted Time Series" OR AB "Interrupted Time Series"  54. TI Regression* OR AB Regression*  55. TI "natural experiment" OR AB "natural experiment"  56. TI "demand analysis" OR AB "demand analysis"  57. TI "discrete choice experiment" OR "discrete choice model*" OR AB "discrete choice experiment" OR "discrete choice model*"  58. TI Economic* OR AB Economic*  59. TI Econometric* OR AB Econometric*  60. TI "quality-adjusted life years" OR AB "quality-adjusted life years"  61. TI "Disability-Adjusted Life Years" OR AB "Disability-Adjusted Life Years"  62. TI cross-sectional analysis OR AB cross-sectional analysis  63. S44 OR S45 OR S46 OR S47 OR S48 OR S49 OR S50 OR S51 OR S52 OR S53 OR S54 OR S55 OR S56 OR S57 OR S58 OR S59 OR S60 OR S61 OR S62  64. S18 AND S32 AND S43 AND S63  65. TI review*  66. S64 AND S65  67. Limiters - Publication Year: 2012-; Publication Type: Journal Article; Human; Language: English |
| **Cochrane Database of Systematic Reviews - 20**   1. MeSH descriptor: [Sugars] this term only 2. (sweeten* or junk):ti,ab,kw 3. MeSH descriptor: [Dietary Sugars] this term only 4. MeSH descriptor: [Beverages] this term only 5. MeSH descriptor: [Carbonated Beverages] this term only 6. #1 or #2 or #3 or #4 or #5 7. MeSH descriptor: [Salts] this term only 8. MeSH descriptor: [Sodium, Dietary] this term only 9. #7 or #8 10. MeSH descriptor: [Trans Fatty Acids] this term only 11. MeSH descriptor: [Dietary Fats] this term only 12. MeSh descriptor: [Fatty Acids] this term only 13. MeSH descriptor: [Fruit] this term only 14. MeSH descriptor: [Fruit and Vegetable Juices] this term only 15. MeSH descriptor: [Vegetables] this term only 16. MeSH descriptor: [Food] this term only 17. (food*):ti,ab,kw 18. MeSH descriptor: [Diet] this term only 19. MeSH descriptor: [Food Supply] this term only 20. (Food growing):ti,ab,kw 21. MeSH descriptor: [Agriculture] this term only 22. MeSH descriptor: [Supermarkets] this term only 23. MeSH descriptor: [Restaurants] this term only 24. MeSH descriptor: [Food Preferences] this term only 25. #6 or #9 or #10 or #11 or #12 or #13 or #14 or #15 or #16 or #17 or #18 or #19 or #20 or #21 or #22 or #23 or #24 26. MeSH descriptor: [Taxes] this term only 27. (tax* or price* or pricing):ti,ab,kw 28. MeSH descriptor: [Government Programs] this term only 29. MeSh descriptor: [Policy] this term only 30. MeSH descriptor: [Government Regulation] this term only 31. MeSH descriptor: [Food, Formulated] this term only 32. (change or changing or decreas* or limit* or modify or modified or new or reduce or reducing or reduction* or reformulat* or restrict* or replac* or increas* or discount or deal* or offer* or ban):ti,ab,kw 33. (subsid*):ti,ab,kw 34. MeSH descriptor: [Advertisement] this term only 35. (market* or adverti* or promot*):ti,ab,kw 36. MeSH descriptor: [Food Labeling] this term only 37. ((food* or nutrition* or diet*) NEAR (facts or information or label* or symbol* or warning*)):ti,ab,kw 38. MeSH descriptor: [Health Promotion] this term only 39. (levy):ti,ab,kw 40. MeSH descriptor: [Nutrition Policy] this term only 41. MeSH descriptor: [Nutritional Requirements] this term only 42. MeSH descriptor: [Financing, Government] this term only 43. MeSH descriptor: [Legislation, Food] this term only 44. (interven*):ti,ab,kw 45. #26 or #27 or #28 or #29 or #30 or #31 or #32 or #33 or #34 or #35 or #36 or #37 or #38 or #39 or #40 or #41 or #42 or #43 or #44 46. MeSH descriptor: [Population] this term only 47. (population):ti,ab,kw 48. MeSH descriptor: [Adult] this term only 49. MeSH descriptor: [Government] this term only 50. (national population):ti,ab,kw 51. MeSH descriptor: [Population Health] this term only 52. MeSH descriptor: [Global Health] this term only 53. MeSH descriptor: [Public Health] this term only 54. (policy maker* or policymaker*):ti,ab,kw 55. (Consumer*):ti,ab,kw 56. (customer*):ti,ab,kw 57. (purchas*):ti,ab,kw 58. (consumption):ti,ab,kw 59. MeSH descriptor: [Food Industry] this term only 60. #46 or #47 or #48 or #49 or #50 or #51 or #52 or #53 or #54 or #55 or #56 or #57 or #58 or #59 61. "economic analysis" 62. macroeconomic* 63. macro-economic* 64. economic NEXT evaluat* 65. (cost*):ti 66. cost NEXT benefit NEXT analys* 67. MeSH descriptor: [Cost-Benefit Analysis] this term only 68. MeSH descriptor: [Costs and Cost Analysis] this term only 69. "cost consequence" 70. "difference in difference" 71. MeSH descriptor: [Interrupted Time Series Analysis] this term only 72. MeSH descriptor: [Regression Analysis] this term only 73. MeSH descriptor: [Models, Theoretical] this term only 74. MeSH descriptor: [Computer Simulation] this term only 75. MeSH descriptor: [Models, statistical] this term only 76. "natural experiment" 77. "demand analysis" 78. "discrete choice model" 79. "discrete choice experiment" 80. MeSH descriptor: [Economics] this term only 81. Econometric* 82. MeSH descriptor: [Models, Economic] this term only 83. MeSH descriptor: [Quality-Adjusted Life Years] this term only 84. MeSH descriptor: [Disability-Adjusted Life Years] this term only 85. MeSH descriptor: [Cross-Sectional Studies] this term only 86. #61 or #62 or #63 or #64 or #65 or #66 or #67 or #68 or #69 or #70 or #71 or #72 or #73 or #74 or #75 or #76 or #77 or #78 or #79 or #80 or #81 or #82 or #83 or #84 or #85 87. #25 and #45 and #60 and #86   88. With Cochrane Library publication date from Jan 2012 to Nov 2024, in Cochrane Reviews |
| **CRD 5**  1 MeSH DESCRIPTOR Sugars EXPLODE ALL TREES  2 (sweeten* or junk)  3 MeSH DESCRIPTOR Dietary Sugars EXPLODE ALL TREES  4 MeSH DESCRIPTOR Beverages EXPLODE ALL TREES  5 MeSH DESCRIPTOR Carbonated Beverages EXPLODE ALL TREES  6 (#1 OR #2 OR #3 OR #4 OR #5)  7 MeSH DESCRIPTOR Salts EXPLODE ALL TREES  8 MeSH DESCRIPTOR Sodium, Dietary EXPLODE ALL TREES  9 (#7 OR #8)  10 MeSH DESCRIPTOR Trans Fatty Acids EXPLODE ALL TREES  11 MeSH DESCRIPTOR Dietary Fats EXPLODE ALL TREES  12 MeSH DESCRIPTOR Fatty Acids EXPLODE ALL TREES  13 MeSH DESCRIPTOR Fruit EXPLODE ALL TREES  14 MeSH DESCRIPTOR Fruit and Vegetable Juices EXPLODE ALL TREES  15 MeSH DESCRIPTOR Vegetables EXPLODE ALL TREES  16 MeSH DESCRIPTOR Food EXPLODE ALL TREES  17 (food*)  18 MeSH DESCRIPTOR Diet  19 MeSH DESCRIPTOR Food Supply  20 (food growing)  21 MeSH DESCRIPTOR Agriculture  22 MeSH DESCRIPTOR Supermarkets EXPLODE ALL TREES  23 MeSH DESCRIPTOR Restaurants  24 MeSH DESCRIPTOR Food Preferences  25 (#6 OR #9 OR #10 OR #11 OR #12 OR #13 OR #14 OR #15 OR #16 OR #17 OR #18 OR #19 OR #20 OR #21 OR #22 OR #23 OR #24)  26 MeSH DESCRIPTOR Taxes  27 (tax* or price* or pricing)  28 MeSH DESCRIPTOR Government Programs  29 MeSH DESCRIPTOR Policy  30 MeSH DESCRIPTOR Government Regulation  31 MeSH DESCRIPTOR Food, Formulated  32 (change or changing or decreas* or limit* or modify or modified or new or reduce or reducing or reduction* or reformulat* or restrict* or replac* or increas* or discount or deal* or offer* or ban)  33 (subsid*)  34 MeSH DESCRIPTOR Advertising  35 (market* or adverti* or promot*)  36 MeSH DESCRIPTOR Food Packaging  37 ((food* or nutrition* or diet*) NEAR (facts or information or label* or symbol* or warning*))  38 MeSH DESCRIPTOR Health Promotion  39 (levy)  40 MeSH DESCRIPTOR Nutrition Policy  41 MeSH DESCRIPTOR Nutritional Requirements  42 MeSH DESCRIPTOR Financing, Government  43 MeSH DESCRIPTOR Legislation, Food  44 (interven*)  45 (#26 OR #27 OR #28 OR #29 OR #30 OR #31 OR #32 OR #33 OR #34 OR #35 OR #36 OR #37 OR #38 OR #39 OR #40 OR #41 OR #42 OR #43 OR #44)  46 MeSH DESCRIPTOR Population  47 (population)  48 MeSH DESCRIPTOR Adult  49 MeSH DESCRIPTOR Government  50 (national population)  51 MeSH DESCRIPTOR Population Health  52 MeSH DESCRIPTOR Global Health  53 MeSH DESCRIPTOR Public Health  54 (policy maker* or policymaker*)  55 (Consumer*)  56 (customer*)  57 (purchas*)  58 (consumption)  59 MeSH DESCRIPTOR Food Industry  60 (#46 OR #47 OR #48 OR #49 OR #50 OR #51 OR #52 OR #53 OR #54 OR #55 OR #56 OR #57 OR #58 OR #59)  61 (economic analysis)  62 (macroeconomic*)  63 (macro-economic*)  64 (economic evaluat*)  65 (cost*):TI  66 (cost benefit analys*)  67 MeSH DESCRIPTOR Cost-Benefit Analysis  68 MeSH DESCRIPTOR Costs and Cost Analysis  69 (cost consequence)  70 (difference in difference)  71 MeSH DESCRIPTOR Interrupted Time Series Analysis  72 MeSH DESCRIPTOR Regression Analysis  73 MeSH DESCRIPTOR Models, Theoretical  74 MeSH DESCRIPTOR Computer Simulation  75 MeSH DESCRIPTOR Models, Statistical  76 (natural experiment)  77 (demand analysis)  78 (discrete choice model*)  79 (discrete choice experiment)  80 MeSH DESCRIPTOR Economics  81 (Econometric*)  82 MeSH DESCRIPTOR Models, Economic  83 MeSH DESCRIPTOR Quality-Adjusted Life Years  84 MeSH DESCRIPTOR Cross-Sectional Studies  85 (#61 OR #62 OR #63 OR #64 OR #65 OR #66 OR #67 OR #68 OR #69 OR #70 OR #71 OR #72 OR #73 OR #74 OR #75 OR #76 OR #77 OR #78 OR #79 OR #80 OR #81 OR #82 OR #83 OR #84 )  86 (#25 AND #45 AND #60 AND #85)  87 (review*):TI  88 #86 AND #87  89 * FROM 2012 TO 2024  90 #88 AND #89 |

**Supplementary Table S3:** **Summary of main review characteristics**

| **Author/year of publication/number of primary studies in the review** | **Intervention focus** | **Aim of the review** | **Study design within primary studies included** | **Effectiveness reported** | **Quality appraisal of primary studies by review authors** |
| --- | --- | --- | --- | --- | --- |
| SUPPLY SIDE INTERVENTIONS | | | | | |
| Dangour et al., 2013 n = 4 | Agricultural policy that directly affect the price of food | To understand how agricultural policies that affect the price of food influence the prevalence of nutrition-related chronic disease in children and adults. | 2 ex post evaluations and 2 ex ante simulations | Lack of evidence on the impact of agricultural price policies on nutrition and health. | Good |
| De Steur et al., 2017 n = 16 | Agricultural reformulation (genetic modification) | To synthesise evidence on consumers’ willingness to pay (WTP) and cost-effectiveness of genetic modified biofortified crops. | Economic evaluations: 1 CBA, 2 CEA, 2 CEA&CBA, and 11 WTP studies | Consumers are WTP 23.9% more for genetically modified biofortified crops.  Cost-effectiveness: USD 7.9–27.8 per DALY in a pessimistic and optimistic scenario, respectively. | N/A |
| Cormick et al., 2021 n = 20 | Calcium fortification | To determine the effectiveness and cost-effectiveness of calcium fortified foods | 15 RCTs, 3 non-randomised studies, and 2 economic evaluations | Calcium fortification leads to a higher calcium intake, and small benefits in children’s height and bone health.  Fortification programmes are cost-saving | Overall quality was low due to lack of blinding and attrition bias. Meta-analysis assessed using the GRADE approach, graded low to moderate for different outcomes. |
| Aguiar et al., 2017 n = 14 | Vitamin D deficiency (VDD) prevention strategies, including supplementation, and food fortification. | To review and critically appraise economic evaluations of population strategies to prevent VDD | Economic evaluations: 6 CUA, 5 CEA, 1 CCA, and 2 CUA&CEA | Paucity of data on VD fortification (insufficient evidence to draw conclusions) | None of the studies fulfilled all Drummond checklist. Lack of rigour in reporting methods, and model inputs and assumptions. |
| DEMAND SIDE INTERVENTIONS | | | | | |
| Thow et al., 2014 n = 38 | Food taxes and subsidies | To estimate the effect of food taxes and subsidies on consumption. | 2 RCTs, 29 modelling, and 7 empirical studies | Taxes/subsidies likely to be effective, however some uncertainty due to heavy reliance on prospective studies rather than observed outcomes. | Majority of modelling studies had high level of uncertainty. |
| Redondo et al., 2018 n = 17 | Sugar-sweetened beverage (SSB) tax | To synthesise evidence from real-world taxes and from other empirical approaches related to the impact of taxes on the consumption, purchase, or sales of SSBs. | 12 virtual or experimental, and 5 naturalistic experimental studies | SSB taxation is effective in reducing SSB purchases. | Evidence was assessed using the CONSORT and TREND statements. Quality was unclear, due to irrelevance of some criteria for food field data. |
| Maniadakis et al., 2013 n = 55 | Tax on SSBs or Foods High In Fat Sugar or Salt (HFSS) | To assess the possible effects of tax policies on consumption of SSBs and HFSS, caloric intake, or weight outcomes. | 22 demand studies, 11 longitudinal studies, 11 cross-sectional studies, 6 modelling studies, 4 experimental studies, 1 cohort study | Effect of tax to curb obesity is doubtful. Limited number of studies report on weight outcomes. Should be cautious because majority of studies transform consumption to weight outcomes based on extrapolation models. | N/A |
| Backholer et al., 2016 n = 11 | Tax on SSBs | To estimate the impact of SSB taxes on beverage purchases and consumption, weight outcomes across socioeconomic groups. | 3 empirical studies, 1 price elasticity estimation of SSB demand, and 7 modelling studies | SSB tax will have greater benefits for lower socioeconomic groups. | Quality assessed using a checklist derived from two recent reviews. 6/11 studies received a score of >=4 points (out of 7). |
| Teng et al., 2019 n = 18 | ‘Real world’ SSB tax | To examine ‘real-world’ SSB tax on beverage purchasing and dietary intake using a meta‐analysis. | 15 ITS, 5 before and after studies, and 2 cross-sectional studies | Based on real‐world evaluations, SSB tax appears to have been effective in reducing SSB purchases and dietary intake. | A study-specific critical appraisal tool was used. Studies were generally high-quality observational studies. |
| Thiboonboon et al., 2024 n=14 | SSB tax | To identify economic evaluations of SSB taxes as a strategy to combat obesity. | Economic evaluations: 4 CEA and 10 CUA | Studies demonstrated that SSB taxes may be effective at reducing obesity, addressing health inequities, and result in healthcare cost savings. However, estimating SSB tax effects was uncertain due to a reliance on indirect evidence that was less robust than evidence from RCTs | N/A |
| Powell et al., 2013 n = 36 | Food and beverage taxes and subsidies | To estimate the recent U.S. studies on the effect of taxes and subsides on food and beverage consumption, and body weight outcomes. | 10 demand studies, 11 longitudinal studies, and 19 cross-sectional studies | Pricing instruments should be considered and evaluated as potential interventions to reduce public health risk. | N/A |
| Pfinder et al., 2020 n = 1 | Taxation of unprocessed sugar or sugar-added foods | To assess the effects of taxation of unprocessed sugar or sugar-added foods in the population on consumption of targeted foods, body weight, and other diet-related health outcomes. | 1 ITS | Limited evidence, uncertain if tax has an effect | Quality assessed using the GRADE approach, which showed very low-certainty evidence. |
| Niebylski et al., 2015 n = 78 | Food subsidies and taxation | To assess the effect of healthy food/beverage subsidies and unhealthy food/beverage taxation. | 3 economic evaluations; 33 modelling; 13 empirical; 10 experimental; and 19 miscellaneous studies | Consistent evidence that tax and subsidy effective at influencing diet | Used the GRADE approach. Studies moderate in quality |
| Liu et al., 2022 n = 15 | Taxation of sugary foods and beverages | To review evidence on the cost effectiveness of taxation policies for sugary foods and beverages. | Economic evaluations: 12 CUA, and 3 CUA&CEA | Sugar tax is a cost-effective policy to reduce the economic burden of excess sugar consumption | Quality of studies was assessed using the Consolidated Health Economic Evaluation Reporting Standards (CHEERS). Overall good quality studies. |
| Lhachimi et al., 2020 n = 2 | Fat tax on foods | To estimate the effects of a fat tax on fat consumption, energy intake, overweight, obesity, and other adverse health outcomes in the general population. | 2 ITS | Uncertain due to low evidence | Quality of studies assessed using GRADE approach, which showed very low level of certainty. |
| Gittelsohn et al., 2017 n = 30 | Pricing policy using taxes and subsidies | To estimate the effect of food-pricing interventions on retail sales and on consumer purchasing and consumption of healthy foods and beverages. | 15 RCTs, 2 NRCTs, 1 experimental study, 7 quasi-experiments, 4 before-and-after studies, and 2 mixed-methods study | Pricing interventions appear to be effective in increasing stocking, sales, purchasing, and consumption of promoted foods and beverages. | The mean score for quality of research measures was 6.9 (standard deviation, 2.0), on a scale of 0 to 10 points. |
| Eyles et al., 2012 n = 32 | Pricing policy using taxes and subsidies | To estimate the association between food pricing strategies and food purchases or intake (consumption)/health and disease outcomes. | 32 modelling studies | Taxes on carbonated drinks and saturated fat and subsidies on fruits and vegetables would be associated with beneficial dietary change. | Low quality of the majority (25/32) of included studies. |
| Epstein et al., 2012 n = 24 | Pricing policies | To investigate the extent to which price changes influence purchases of targeted and nontargeted foods. | 24 experimental studies | Price changes modify purchases of targeted foods, but research on the overall nutritional quality of purchases is mixed because of substitution effects. | N/A |
| Cornelsen et al., 2015 n = 78 | Pricing policies (taxes, price and subsidies) | To review the global evidence on food price elasticities. | 78 price elasticity estimates generated from demand studies | The own-price effects were found to be significant. Cross-price effects were more varied and depended on country income levels. | Quality was assessed based on the description of data, methods and food groups sufficient to replicate the study. 26/78 studies covering 1963 estimates were classified as ‘replicable’. |
| Afshin et al., 2017 n = 30 | Pricing policies (taxes, price and subsidies) | To quantify the prospective effects of a change in food price on consumption. | 23 intervention studies (7 randomised, 16 nonrandomised), 7 prospective cohorts | Results supported efficacy of taxes and subsidies to support healthy eating | Quality was assessed based on 5 criteria: study design, assessment of exposure, assessment of outcome, control for confounding, and evidence of selection bias.  20 lower quality and 10 higher quality studies. |
| Alagiyawanna et al., 2015 n = 18 | Taxes and subsidies | To study the behavioural and health outcomes of implemented food and beverage fiscal interventions. | 1 natural experiment, 2 ITS, 9 cross-sectional studies, 2 longitudinal studies, 2 controlled before and after studies, 1 uncontrolled before and after study, and 1 ecological study | Fiscal interventions on foods can influence consumption of taxed and subsidised foods and consequently have potential to improve health. But confidence low. | Quality assessed using the Evaluation of Public Health Practice Projects (EPHPP). 9 studies were weak, 7 studies were moderate, 2 studies were strong. |
| An, 2013 n = 20 | Healthy food subsidies | To estimate the effect of monetary subsidies in promoting healthier food purchases and consumption. | 9 RCTs, 8 pre-post studies, and 3 cohort studies | Subsidies tend to be effective in increasing the purchase/consumption of targeted products. | Quality assessed using method from existing publication. On average, studies met 6/10 quality criteria, but the studies differed substantially across criteria. |
| Pineda et al., 2024 n = 20 | Tax on HFSS | To assess the impact of HFSS food tax on sales and consumption of HFSS foods, while considering potential impacts on inequalities. | 13 observational studies of implemented taxes and 7 experimental studies of hypothetical taxes | Taxes were effective in decreasing sales, or purchases, and intake of taxed HFSS foods, especially when combined with subsidies on healthy foods. | Overall quality assessed using the GRADE approach, which showed moderate level of certainty for the consumption and sales and low level for the effect on obesity or BMI. |
| Wyse et al., 2021 n = 11 | Mix of online interventions (nudge, labelling, education, tax, and price changes) to encourage healthier purchasing | To explore the effectiveness of dietary interventions delivered using real-world online food ordering systems. | 6 RCTs, 2 crossover RCTs, 2 cluster RCTs, and 1 non-randomised controlled trials | Meta analysis - online interventions are effective in reducing the energy content in purchasing | N/A |
| Fattore et al., 2014 n = 36 | Voluntary interventions (labelling and counselling) promoting low-fat diets | To review economic evaluation studies on interventions aimed at promoting voluntary dietary improvements through reduction of fat intake. | Economic evaluations: 13 CUA, 11 CEA, 5 CBA, 4 CUA&CEA, 1 CEA&CBA, 2 CUA&CBA | Inconclusive results of cost effectiveness. There was limited experimental evidence. Design for economic evaluation requires improvement. | Used the CHEC-list. Rating was low. |
| DEMAND AND SUPPLY SIDE INTERVENTIONS | | | | | |
| Hyseni et al., 2017 n = 70 | Salt Reduction: Including reformulation, dietary advice, tax, school-based intervention, and labelling | To synthesise evidence on various salt reduction interventions. | 49 empirical and 21 modelled-based primary studies | Comprehensive strategies involving multiple components (reformulation, food labelling and media campaigns) and including upstream policies such as mandatory reformulation achieve larger reductions in population-wide salt consumption, relative to downstream individual- focussed interventions. | Quality was assessed using the National Heart, Lung and Blood Institute (NHLBI) tool. Modelling studies were assessed using a different tool adapted from an existing literature.  Quality was variable. |
| Schorling et al., 2017 n = 14 | Salt reduction: mix of interventions to reduce salt and risk of hypertension and cardiovascular disease | To examine whether different approaches of population-wide and targeted salt reduction interventions are cost-effective. | Economic evaluations:  8 CUA, 3 CEA, and 3 CUA&CEA | Population-wide salt reduction interventions could be cost-effective in prevention of hypertension and CVD in OECD member countries. | Quality was assessed using the British Medical Journal checklist. No study fulfilled all 36 items checklist items. |
| McLaren et al., 2016 n = 17 | Salt reduction: Mix of interventions to reduce salt intake (reformulation, food procurement policy, labelling, restrictions on marketing to children) | To assess the impact of population-level interventions for dietary sodium reduction in government jurisdictions worldwide | 14 uncontrolled pre-post, and 1 open-cohort design. | Population-level interventions for sodium reduction have potential to reduce salt, particularly if they are multi-component (more than one intervention activity) and incorporate interventions of a structural nature (e.g. reformulation). | Quality was assessed using adapted version of the Cochrane risk of bias tool and the GRADE approach.  Overall quality was very low due to high risk of bias. |
| Hope et al., 2017 n = 14 | Salt reduction: Mix of interventions to reduce salt in population | To summarise evidence describing the cost-effectiveness of population-based interventions targeting sodium reduction. | Economic evaluations: 14 CUA | Population-based salt reduction interventions are likely to be cost effective or cost saving. However, given the reliance on modelling, there is a need for the effectiveness of new interventions to be evaluated in the field using strong study designs and parallel economic evaluations (need better evidence). | Quality was assessed using CHEERS and was generally good. 8/14 were excellent, 5 were very good and 1 was good. |
| Barberio et al., 2017 n=41 | Salt reduction: Mix of interventions including reformulation, tax, food procurement policy, restricting on marketing to children, labelling and education. | To assess the impact of population-level interventions for dietary sodium reduction in government jurisdictions worldwide. | 14 uncontrolled pre-post design and 1 open cohort design | Population-level dietary sodium reduction initiatives have the potential to reduce dietary salt intake, especially if they are multicomponent and incorporate intervention activities of a structural nature. | Quality was assessed using an adapted version of the Cochrane risk of bias tool and GRADE The overall quality was very low due to high risk of bias, reflecting the observational nature of the research and the use of an uncontrolled study design. |
| von Philipsborn et al., 2019 n = 58 | Mix of physical or social environment interventions to reduce SSB | To assess the effects of environmental interventions (excluding taxation) on the consumption of SSB, diet-related anthropometric measures and health outcomes. | 22 RCTs, 3 NRCTs, 14 CBA studies, and 19 ITS studies | Effective, scalable interventions exist, addressing SSB consumption at a population level. | Overall quality was assessed using GRADE. The overall confidence in the certainty of effects was low to moderate because most of the studies used non-randomised designs. |
| Alcaraz et al., 2021 n = 40 | SSB reduction: Interventions to reduce SSB including tax, school environment, advertising, labelling and subsidies | To identify the best available epidemiological or decision models applicable to assess SSB related consumption | 40 modelling studies | Cost effectiveness was inconclusive. Wide range of modelling methods applied | N/A |
| Tran et al., 2021 n = 8 | Mix of health-promoting food retail-based interventions | To assess the evidence on cost-effectiveness of food retail interventions to improve diet-related health outcomes | Economic evaluations: 4 CEA, 3 CUA, and 1 CEA&CUA | The cost-effectiveness of retail-based health-promoting interventions is inconclusive | Quality assessed using CHEERS checklist. Compliance ranged from 65% to 96%. |
| Olm et al., 2020 n = 21 | Obesity policy: including nutritional interventions (food habit interventions or tax) - impact on obesity, T2DM costs | To critically assess nutritional interventions for their impact on healthcare costs to community-dwelling individuals regarding type 2 diabetes or obesity or both. | 13 cost of illness studies,  Economic evaluations: 2 CUA, 1 CEA, 3 CEA&CUAs, 2 burden of disease & cost of illness studies | Obesity policy could lead to cost savings and improve health outcomes when compared with current practice. | Quality was assessed using CHEERS checklist. Overall, the 21 included studies were of very good quality but the risk of bias associated with the conclusion was high because all included studies were model-based. |
| Hillier-Brown et al., 2017 n = 30 | Mix of interventions to promote healthier ready meals including legislation restriction, menu changes, price changes, and labelling, | To review the literature on the impact of interventions to promote healthier ready-to-eat meals (to eat in, to take away or to be delivered) sold by specific food outlets accessible to the general public. | 19 repeat cross-sectional studies, 5 cohort studies, 2 controlled before-and-after studies, and 4 controlled trials | Interventions to promote healthier ready-to-eat meals sold by food outlets should restrict choice or guide choice through incentives/disincentives. Public health policies and practice that simply involve providing information are unlikely to be effective. | Quality was assessed using the Effective Public Health Practice Project Quality Assessment Tool. The studies were of low to moderate quality, with few high-quality designs. |
| Emmert-Fees et al., 2021 n= 56 | Mix of population-based dietary policies including reformulation, taxation, and labelling; the most common targets were salt/sodium, sugar-sweetened beverages, and F&V. | To map and critically appraise economic evaluations of population-based dietary policies using simulation models. | Economic evaluations: 17 CUA, 13 CEA, and 26 CUA&CEA [markov 35/36, microsimulation 11/56 and comparative risk assessment models 7/56 ] | Most dietary policies (103/136) were evaluated as cost-saving independent of the applied costing perspective. | Quality was assessed using revised CHEERS checklist. Approximately half of the studies (29/56) fulfilled 90% or more of all quality criteria. |

*CBA: Cost-benefit analysis; CEA: Cost-effectiveness analysis; CUA: Cost-utility analysis; ITS: interrupted time series analysis
